# *M* ^+^ *M* ^3+^_2_As(HAsO_4_)_6_ (*M* ^+^ *M* ^3+^ = TlGa, CsGa, CsAl): three new metal arsenates containing AsO_6_ octa­hedra

**DOI:** 10.1107/S2056989018010721

**Published:** 2018-07-27

**Authors:** Karolina Schwendtner, Uwe Kolitsch

**Affiliations:** a Institute for Chemical Technology and Analytics, Division of Structural Chemistry, TU Wien, Getreidemarkt 9/164-SC, 1060 Wien, Austria; b Naturhistorisches Museum, Burgring 7, 1010 Wien, Austria; c Institut für Mineralogie und Kristallographie, Universität Wien, Althanstrasse 14, 1090 Wien, Austria

**Keywords:** TlGa_2_As(HAsO_4_)_6_, CsGa_2_As(HAsO_4_)_6_, CsAl_2_As(HAsO_4_)_6_, AsO_6_, AsO_6_ octa­hedra, arsenate, crystal structure

## Abstract

The crystal structures of hydro­thermally synthesized (*T* = 493 K, 7 d) thallium(I) digallium arsenic(V) hexa­kis­[hydrogenarsenate(V)], TlGa_2_As(HAsO_4_)_6_, caes­ium digallium arsenic(V) hexa­kis­[hydrogenarsenate(V)], CsGa_2_As(HAsO_4_)_6_, and caesium dialuminium arsenic(V) hexa­kis­[hydrogenarsenate(V)], CsAl_2_As(HAsO_4_)_6_, were solved by single-crystal X-ray diffraction. The three compounds are isotypic and adopt the structure type of RbAl_2_As(HAsO_4_)_6_ (*R*  *c*), which itself represents a modification of the RbFe(HPO_4_)_2_ structure type and consists of a tetra­hedral–octa­hedral framework in which the slightly disordered *M* ^+^ cations are located in channels. The three new compounds contain AsO_6_ octa­hedra assuming the topological role of *M* ^3+^O_6_ octa­hedra. The As—O bond lengths are among the shortest As—O bond lengths known so far in AsO_6_ octa­hedra.

## Abstract 2

The crystal structures of hydro­thermally synthesized TlGa_2_As(HAsO_4_)_6_, CsGa_2_As(HAsO_4_)_6_ and CsAl_2_As(HAsO_4_)_6_ were solved by single-crystal X-ray diffraction. They all crystallize in the RbAl_2_As(HAsO_4_)_6_ structure type (*R* 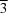
c). The three compounds contain AsO_6_ octa­hedra assuming the topological role of *M* ^3+^O_6_ octa­hedra.

## Chemical context   

Compounds with mixed tetra­hedral–octa­hedral (T–O) framework structures feature a broad range of different atomic arrangements, resulting in topologies with various inter­esting properties such as ion exchange (Masquelier *et al.*, 1996 ▸) and ion conductivity (Chouchene *et al.*, 2017 ▸), as well as unusual piezoelectric (Ren *et al.*, 2015 ▸), magnetic (Ouerfelli *et al.*, 2007 ▸) or non-linear optical features (frequency doubling) (Sun *et al.*, 2017 ▸).

The three new compounds were obtained during an extensive experimental study of the system *M* ^+^–*M* ^3+^–O–(H)–As^5+^ (*M* ^+^ = Li, Na, K, Rb, Cs, Ag, Tl, NH_4_; *M* ^3+^ = Al, Ga, In, Sc, Fe, Cr, Tl). This system was found to contain representatives of a large variety of new structure types (Schwendtner & Kolitsch, 2004 ▸, 2005 ▸, 2007*a* ▸,*b* ▸,*c* ▸, 2017*a* ▸, 2018*a* ▸; Schwendtner, 2006 ▸).

Among the many different structure types found during our study, one atomic arrangement, the RbFe(HPO_4_)_2_-type (Lii & Wu, 1994 ▸; *R* 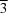 *c*), and relatives thereof (Schwendtner & Kolitsch, 2018*a* ▸) was observed to show a large crystal–chemical flexibility that allows the incorporation of a wide variety of cations. The three title compounds, TlGa_2_As(H­AsO_4_)_6_, CsGa_2_As(HAsO_4_)_6_ and CsAl_2_As(HAsO_4_)_6_ are further representatives of one of these recently described variations of the RbFe(HPO_4_)_2_ type, *viz*. the RbAl_2_As(H­AsO_4_)_6_ type (Schwendtner & Kolitsch, 2018*a* ▸). It also crystallizes in *R* 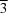 *c* and up to now members with RbAl and CsFe (Schwendtner & Kolitsch, 2018*a* ▸) and RbGa (Schwendtner & Kolitsch, 2018*c* ▸) as *M* ^+^ *M* ^3+^ cation combinations are known (Table 1 ▸). While all previously known *M* ^+^ *M* ^3+^ combinations adopting this structure type also have representatives adopting the RbFe(HPO_4_)_2_ type, this is not the case for the three new members.

The title compounds are rare examples of compounds containing AsO_6_ octa­hedra. According to our review article, only about 3% of all reported arsenates(V) contain AsO_6_ polyhedra (Schwendtner & Kolitsch, 2007*a* ▸). Presently (Schwendtner & Kolitsch, 2018*a* ▸), 41 inorganic compounds containing As in an octa­hedral coordination are known, including the recently published RbGa_2_As(HAsO_4_)_6_ (Schwendtner & Kolitsch, 2018*c* ▸) and the three new compounds of this study. While no arsenates(V) containing both Tl and Ga are known in the ICSD (FIZ, 2018 ▸) so far, there are two arsenates(V) containing both Cs and Ga, namely Cs_2_Ga_3_(As_3_O_10_)(AsO_4_)_2_ (Lin & Lii, 1996 ▸) and CsGa(H_1.5_AsO_4_)_2_(H_2_AsO_4_) (Schwendtner & Kolitsch, 2005 ▸), and one arsenate(V) containing both Cs and Al, CsAl(H_2_AsO_4_)(HAsO_4_) (Schwendtner & Kolitsch, 2007*b* ▸). In addition to the crystal structures contained in the ICSD, an indexed powder diffraction pattern of the diarsenate CsAlAs_2_O_7_ has been published (Boughzala & Jouini, 1992 ▸).

## Structural commentary   

**Figure 1:**
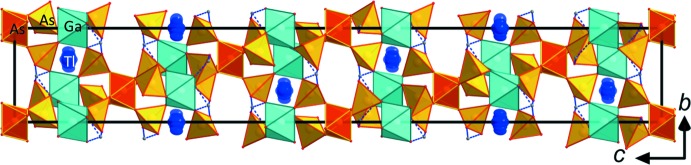
Structure drawing of the framework structure of TlGa_2_As(HAsO_4_)_6_ viewed along *a.* The unit cell is outlined.

**Figure 2:**
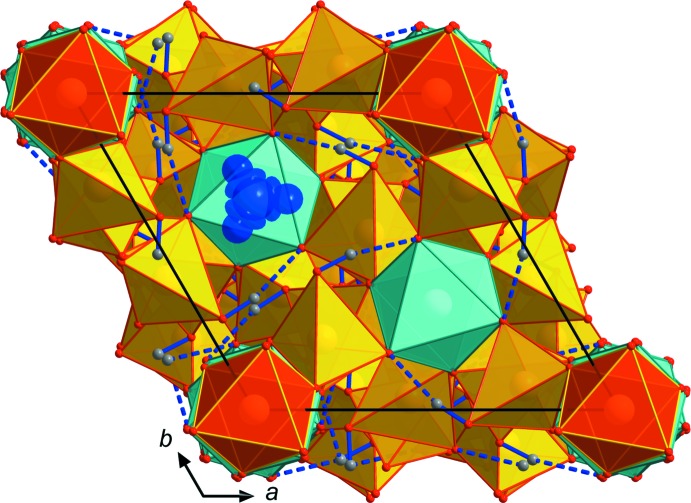
Structure drawing of TlGa_2_As(HAsO_4_)_6_ viewed along *c*. Red octa­hedra = AsO_6_, blue–green octa­hedra = GaO_6_, yellow tetra­hedra = AsO_4_; hydrogen atoms are shown as small grey spheres. Hydrogen bonds are shown as blue lines (solid for *D*—H and dotted for H⋯*A*). For the three disordered Tl positions, the displacement parameters are drawn at the 80% probability level. The unit cell is outlined.

**Figure 3:**
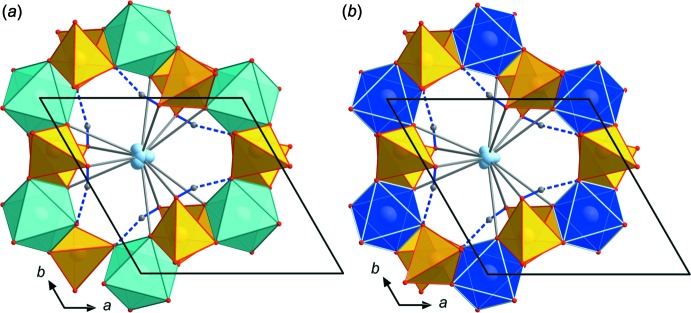
Comparison of the Cs atom disorder, Cs—O bonds and hydrogen-bonding scheme in (*a*) CsGa_2_As(HAsO_4_)_6_ and (*b*) CsAl_2_As(HAsO_4_)_6_, viewed along *c*. Displacement ellipsoids for Cs are drawn at the 80% probability level.

The three title compounds are isotypic and new representatives of the RbAl_2_As(HAsO_4_)_6_-structure type (*R* 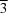 *c;* Schwendtner & Kolitsch, 2018*a* ▸), which is a recently described variation of the RbFe(HPO_4_)_2_ structure type (*R* 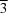 *c;* Lii & Wu, 1994 ▸) and closely related to the following two structure types: (NH_4_)Fe(HPO_4_)_2_ (*P* 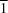; Yakubovich, 1993 ▸) and RbAl(HAsO_4_)_2_ (*R*32; Schwendtner & Kolitsch, 2018*a* ▸). The reader is referred to our latest papers for a detailed discussion of the four related structure types (Schwendtner & Kolitsch, 2018*a* ▸) and a review of compounds crystallizing in the RbFe(HPO_4_)_2_ and (NH_4_)Fe(HPO_4_)_2_ structure types (Schwendtner & Kolitsch, 2018*b* ▸). All of these modifications share a basic tetra­hedral–octa­hedral framework structure featuring inter­penetrating channels, which host the *M* ^+^ cations (Figs. 1 ▸, 2 ▸). The fundamental building unit in all these structure types contains *M* ^3+^O_6_ octa­hedra, which are connected *via* their six corners to six protonated AsO_4_ tetra­hedra, thereby forming an *M* ^3+^As_6_O_24_ unit (Fig. 3 ▸). These units are in turn connected *via* three corners to other *M* ^3+^O_6_ octa­hedra. The free, protonated corner of each AsO_4_ tetra­hedron forms a hydrogen bond to the neighbouring *M* ^3+^As_6_O_24_ group (Table 2 ▸). The *M* ^3+^As_6_O_24_ units are arranged in layers perpendicular to the *c*_hex_ axis (Fig. 2 ▸). When compared to the RbFe(HPO_4_)_2_ structure type, in TlGa_2_As(HAsO_4_)_6_, CsGa_2_As(HAsO_4_)_6_ and CsAl_2_As(HAsO_4_)_6_ one third of all *M* ^3+^ cations are replaced by As^5+^. This requires that two thirds of all *M* ^+^ cations are omitted to achieve charge balance.

Like many other and all isotypic compounds containing AsO_6_ octa­hedra, the three title compounds were grown by ‘dry’ hydro­thermal techniques (using arsenic acid without the addition of water). The extreme abundance of As during the synthesis and the formation of a melt of arsenic acid promotes the octa­hedral coordination of As. As a result of the smaller ionic radius of As^5+^ this substitution also has an effect on the unit-cell parameters (Table 3 ▸) and the pore diameter. While the lengths of the *c* axis of all so far known RbFe(HPO_4_)_2_-type arsenates range from 52.87 (1) to 56.99 (1) Å (Schwendtner and Kolitsch, 2018*b* ▸) and correlate well with the sizes of the involved *M* ^+^ and *M* ^3+^ cations, the length of this axis in the RbAl_2_As(HAsO_4_)_6_-type compounds is much smaller and the range of lengths much narrower [50.17 (1)–50.94 (1) Å, see Table 1 ▸]. The *c* unit-cell parameter correlates with the size of the involved *M* ^3+^ cation, while the *M* ^+^ cations seem to show a negative correlation with the *c* parameter (Table 1 ▸). The unit-cell parameters *a* and *V* correlate well with the size of both cations, but the influence of the *M* ^+^ cations is stronger. It seems that in order to incorporate the small AsO_6_ octa­hedron in the structure the cell widens along the *a* axis to incorporate the large *M* ^+^ cations and is strongly compressed along *c*. This effect is also visible in the hydrogen bonds that are very strong in the RbFe(HPO_4_)_2_-type arsenates with *D*—H⋯*A* bond lengths ranging from 2.598 (2) to 2.634 (2) Å, while for the RbAl_2_As(HAsO_4_)_6_-type arsenates they range from 2.727 (2) to 2.7481 (19) Å (Schwendtner & Kolitsch, 2017*b* ▸, 2018*a* ▸,*b* ▸,*c* ▸; this paper).

In all three title compounds, the *M* ^+^ cations are 12-coord­in­ated (Figs. 3 ▸, 4 ▸), and the average *M* ^+^—O bond lengths (Table 3 ▸) are longer than the average bond lengths of *M* ^+^O_12_ polyhedra of 3.377 Å for Cs (Gagné & Hawthorne, 2016 ▸) and 3.195 Å for Tl^+^ (Gagné & Hawthorne, 2018 ▸), thus leading to rather low bond-valence sums (BVSs) (Gagné & Hawthorne, 2015 ▸) of only 0.46–0.75 valence units (v.u.). The lowest BVS sum was found for ^[12]^Tl, which shows an extremely long average Tl—O bond length (3.439 Å), considerably longer than the longest previously reported value in the literature, 3.304 Å (Gagné & Hawthorne, 2018 ▸). Considering the positions of the disordered Tl-atom positions, the BVSs would increase to 0.54 (Tl1*B*) and 0.68 v.u. (Tl1*C*).

**Figure 4:**
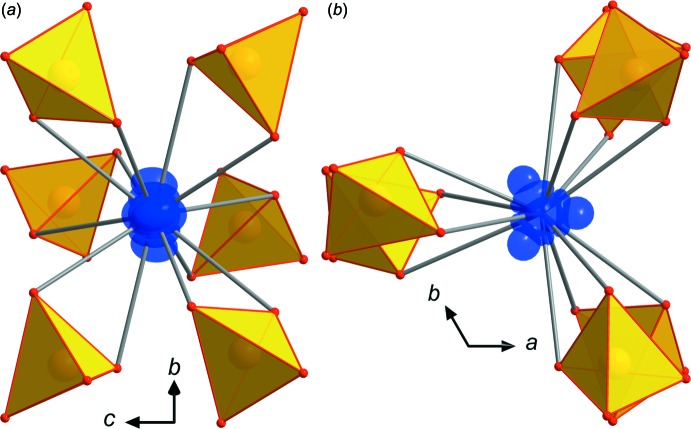
Tl positional disorder and Tl—O bonding scheme for TlGa_2_As(HAsO_4_)_6_ viewed along *a* (*a*) and *c* (*b*). Displacement ellipsoids for Tl are drawn at the 80% probability level.

These loose bondings lead to considerable positional disorder of the *M* ^+^ cations in their hosting voids, which were modelled by two overlapping Cs positions between 0.15 (2) and 0.25 (5) Å apart (Fig. 3 ▸). The main Cs-atom electron densities with 62 and 72% for the Al- and Ga-containing compounds, respectively, are located on the central position Cs1*A*. For the Tl compound, only 33% of the electron-density distribution is explained by the central Tl1*A* position, 36% by the next nearest position Tl1*B* 0.407 (7) Å away and 27% by the furthest Tl1*C* position 0.766 (9) Å apart from the central position – so three positions for Tl were needed to get a good fit explaining the electron-density distribution (Fig. 4 ▸). The effect of slightly disordered *M* ^+^ cations in this type of compound is well known and was found for most of the previously cited compounds crystallizing in these related structure types; in TlGa_2_As(HAsO_4_)_6_ the positional disorder shows its most extreme form, probably as a result of the influence of the lone electron pair on the Tl^+^ cation.

In contrast to the underbonded *M* ^+^ cations the AsO_6_ octa­hedra are overbonded. The six As—O distances in each of these isotypic compounds are identical and among the shortest of all known AsO_6_-containing compounds. TlGa_2_As(HAsO_4_)_6_ shows the shortest average ^[6]^As—O distance known so far of 1.806 Å, leading to rather high BVSs of 5.34 v.u. (after Gagné & Hawthorne, 2015 ▸). The grand mean As—O bond distance in AsO_6_ octa­hedra in inorganic compounds is 1.830 (2) Å according to Schwendtner & Kolitsch (2007*a* ▸); this value was determined from 33 AsO_6_ octa­hedra of 31 compounds. Gagné & Hawthorne (2018 ▸) determined an identical, but less precise, value of 1.83 (3) Å, based on only 13 AsO_6_ octa­hedra in AsO_6_-containing compounds.

A further, indirect effect of the substituting AsO_6_ octa­hedra is a notable change in the As—O distances of the protonated AsO_4_ tetra­hedra. The average As—O distance in these AsO_4_ tetra­hedra, with values between 1.687 and 1.688 Å, is slightly larger in all three compounds than the statistical average of 1.686 (10) Å (Schwendtner, 2008 ▸). The BVSs (Gagné & Hawthorne, 2015 ▸) are close to ideal values (4.98–5.00 v.u.). The AsO_4_ tetra­hedra have two short bond lengths to connected *M* ^3+^O_6_ octa­hedra, but the ^[4]^As—O bond length of the O atom shared with the AsO_6_ octa­hedra is elongated (Table 3 ▸) because of ^[4]^As—O—^[6]^As repulsion. The ^[4]^As—O⋯H bond is therefore shorter than the average distance of As—O⋯H bonds in HAsO_4_ groups [1.72 (3) Å; Schwendtner, 2008 ▸].

The average *M* ^3+^—O bond lengths of the octa­hedrally coordinated Ga cations (1.963–1.965 Å) and Al cations (1.895 Å) are slightly shorter than the grand mean averages of 1.978 (17) and 1.903 (14) Å for ^[6]^Ga—O and ^[6]^Al—O, respectively (Gagné & Hawthorne, 2018 ▸), explaining the slightly higher corresponding BVSs of 3.07 to 3.11 v.u.

## Synthesis and crystallization   

The compounds were grown by hydro­thermal synthesis at 493 K (7 d, autogeneous pressure, slow furnace cooling) using Teflon-lined stainless steel autoclaves with an approximate filling volume of 2 cm^3^. Reagent-grade Cs_2_CO_3_, Tl_2_CO_3_, Ga_2_O_3_, Al_2_O_3_ and H_3_AsO_4_·0.5H_2_O were used as starting reagents in approximate volume ratios of *M* ^+^:*M* ^3+^:As of 1:1:3, 1:2:4 and 1:1:2 for the TlGa-, CsGa- and CsAl-synthesis batches, respectively. No additional water was added and arsenic acid was present in excess to promote the growth of crystals from a melt or even vapor of arsenic acid under extremely acidic conditions. All three compounds formed large, colourless, pseudo-octa­hedral crystals, TlGa_2_As(HAsO_4_)_6_ was accompanied by colourless, acicular-to-prismatic crystals of Ga(H_2_AsO_4_)(H_2_As_2_O_7_) (Schwendtner & Kolitsch, 2017*a* ▸). All crystals were extracted mechanically and not further washed; they are slightly hygroscopic and decompose slowly over a period of several years.

## Refinement   

Crystal data, data collection and structure refinement details are summarized in Table 4 ▸.

For reasons of comparison, the coordinates of RbAl_2_As(HAsO_4_)_6_ (Schwendtner & Kolitsch, 2018*a* ▸) were used for the refinement. These coordinates are also comparable to the related RbFe(HPO_4_)_2_ structure type (*R* 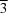 *c;* Lii & Wu, 1994 ▸). In all compounds, O—H bonds were restrained to 0.9±0.02 Å. During the last refinement steps, residual electron-density peaks of up to 5.54 e Å^−3^ were located close to the *M* ^+^ sites, suggesting irregular displacement parameters and split positions, similar to what was found for many other RbFe(HPO_4_)_2_-type compounds and relatives thereof (Lesage *et al.*, 2007 ▸; Schwendtner and Kolitsch, 2018*a* ▸,*b* ▸). Therefore, a further position *M* ^+^1*B* was included in the refinements, which refined to low occupancies and led to considerable decreases in the *R* factors and weight parameters for all compounds.

This, however, was not satisfactory for TlGa_2_As(HAsO_4_)_6_, where it led to negative electron densities of −2.3 e Å^−3^ at the centre of the Tl1*A* position, probably an effect of the lone electron pair. Therefore, the Tl atoms were again removed from the model and the three highest residual electron densities from the difference-Fourier map were then refined simultaneously as Tl1*a*, Tl1*b* and Tl1*c*. This led to a much better fit explaining the disordered electron density. The refined bulk occupancies on the disordered *M* ^+^ positions in all compounds were very close to 1, but a restraint was still set in all cases to give a total occupancy of 1.00. The final residual electron densities in all title compounds are < 1 e Å^−3^.

## Supplementary Material

Crystal structure: contains datablock(s) CsAl2AsHAsO46, CsGa2AsHAsO46, TlGa2AsHAsO46. DOI: 10.1107/S2056989018010721/pj2056sup1.cif

Structure factors: contains datablock(s) CsAl2AsHAsO46. DOI: 10.1107/S2056989018010721/pj2056CsAl2AsHAsO46sup2.hkl

Structure factors: contains datablock(s) CsGa2AsHAsO46. DOI: 10.1107/S2056989018010721/pj2056CsGa2AsHAsO46sup3.hkl

Structure factors: contains datablock(s) TlGa2AsHAsO46. DOI: 10.1107/S2056989018010721/pj2056TlGa2AsHAsO46sup4.hkl

CCDC references: 1857884, 1857883, 1857882

Additional supporting information:  crystallographic information; 3D view; checkCIF report

## Figures and Tables

**Table 1:** Comparison of unit-cell parameters (Å, Å^3^) for the six known isotypic compounds

Compound	*a*	*c*	*V*
TlGa_2_As(HAsO_4_)_6_	8.484 (1)	50.724 (11)	3161.9 (10)
RbGa_2_As(HAsO_4_)_6_	8.491 (1)	50.697 (11)	3165.4 (10)
CsGa_2_As(HAsO_4_)_6_	8.520 (1)	50.608 (11)	3181.4 (10)
RbAl_2_As(HAsO_4_)_6_	8.410 (1)	50.287 (11)	3080.2 (10)
CsAl_2_As(HAsO_4_)_6_	8.439 (1)	50.169 (11)	3094.2 (10)
CsFe_2_As(HAsO_4_)_6_	8.582 (1)	50.942 (11)	3249.3 (10)

**Table 2:** Hydrogen-bond geometry (Å, °) for CsAl_2_As(HAsO_4_)_6_, CsGa_2_As(HAsO_4_)_6_ and TlGa_2_As(HAsO_4_)_6_

	*D*—H⋯*A*	*D*—H	H⋯*A*	*D*⋯*A*	*D*—H⋯*A*
CsAl_2_As(HAsO_4_)_6_	O3—H⋯O4^xiv^	0.875 (19)	1.94 (2)	2.7321 (18)	150 (3)
CsGa_2_As(HAsO_4_)_6_	O3—H⋯O4^xiv^	0.861 (18)	1.93 (2)	2.727 (2)	154 (3)
TlGa_2_As(HAsO_4_)_6_	O3—H⋯O4^xiv^	0.871 (19)	1.94 (3)	2.728 (2)	150 (4)

Symmetry code: (xiv) *y*, *x* − 1, −*z* + 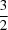.

**Table 3:** Comparison of selected bond lengths (Å) and BVSs*^*a*^* for CsAl_2_As(HAsO_4_)_6_, CsGa_2_As(HAsO_4_)_6_ and TlGa_2_As(HAsO_4_)_6_

	**CsAl_2_As(HAsO_4_)_6_**	**CsGa_2_As(HAsO_4_)_6_**	**TlGa_2_As(HAsO_4_)_6_**
*M* ^+^1*A*—O2 (6×)	3.4707 (12)	3.4719 (15)	3.4419 (15)
*M* ^+^1*A*—O3 (6×)	3.4066 (16)	3.4829 (19)	3.4358 (19)
**<*M*^+^1*A*—O>/BVS**	**3.439/0.75**	**3.477/0.68**	**3.439/0.46**
*M* ^3+^—O2 (3×)	1.8933 (13)	1.9612 (15)	1.9648 (14)
*M* ^3+^—O4 (3×)	1.8963 (12)	1.9679 (15)	1.9609 (14)
**<*M*^3+^—O>/BVS**	**1.895/3.07**	**1.965/3.09**	**1.963/3.11**
^[6]^As—O (6×)	1.8104 (11)	1.8109 (14)	1.8062 (14)
**<^[6]^As—O>/BVS**	**1.810/5.27**	**1.811/5.27**	**1.806/5.34**
^[4]^As—O1	1.7094 (12)	1.7089 (14)	1.7094 (14)
^[4]^As—O2	1.6641 (11)	1.6646 (14)	1.6641 (14)
^[4]^As—O4	1.6639 (12)	1.6670 (15)	1.6672 (14)
^[4]^As—O3(H)	1.7108 (13)	1.7125 (17)	1.7115 (16)
**<^[4]^As—O>/BVS**	**1.687/5.00**	**1.688/4.98**	**1.688/4.99**

Note: (*a*) Gagné & Hawthorne (2015 ▸).

**Table 4:** Experimental details

	CsAl_2_As(HAsO_4_)_6_	CsGa_2_As(HAsO_4_)_6_	TlGa_2_As(HAsO_4_)_6_
Crystal data			
*M*_r_	1101.36	1186.84	1258.30
Crystal system, space group	Trigonal, *R* 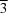 *c*:*H*	Trigonal, *R* 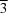 *c*:*H*	Trigonal, *R* 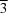 *c*:*H*
Temperature (K)	293	293	293
*a*, *c* (Å)	8.439 (1), 50.169 (11)	8.5199 (10), 50.608 (11)	8.484 (1), 50.724 (11)
*V* (Å^3^)	3094.2 (10)	3181.4 (10)	3161.9 (10)
*Z*	6	6	6
Radiation type	Mo *K*α	Mo *K*α	Mo *K*α
μ (mm^−1^)	13.14	15.18	21.18
Crystal size (mm)	0.08 × 0.07 × 0.06	0.07 × 0.07 × 0.07	0.08 × 0.07 × 0.05

Data collection			
Diffractometer	Nonius KappaCCD single-crystal four-circle	Nonius KappaCCD single-crystal four-circle	Nonius KappaCCD single-crystal four-circle
Absorption correction	Multi-scan (*HKL* *SCALEPACK*; Otwinowski *et al.*, 2003 ▸)	Multi-scan (*HKL* *SCALEPACK*; Otwinowski *et al.*, 2003 ▸)	Multi-scan *HKL* *SCALEPACK* (Otwinowski *et al.*, 2003 ▸)
*T*_min_, *T*_max_	0.420, 0.506	0.416, 0.416	0.200, 0.347
No. of measured, independent and observed [*I* > 2σ(*I*)] reflections	4584, 1259, 1153	4712, 1293, 1134	4682, 1285, 1129
*R*_int_	0.014	0.018	0.024
(sin θ/λ)_max_ (Å^−1^)	0.757	0.757	0.757

Refinement			
*R*[*F* ^2^ > 2σ(*F* ^2^)], *wR*(*F* ^2^), *S*	0.015, 0.034, 1.11	0.018, 0.041, 1.16	0.018, 0.041, 1.10
No. of reflections	1259	1293	1285
No. of parameters	65	65	71
No. of restraints	2	2	2
H-atom treatment	All H-atom parameters refined	All H-atom parameters refined	All H-atom parameters refined
Δρ_max_, Δρ_min_ (e Å^−3^)	0.61, −0.45	0.86, −0.73	0.74, −0.73

Computer programs: *COLLECT* (Nonius, 2003 ▸), *HKL* *DENZO*, *SCALEPACK* (Otwinowski *et al.*, 2003 ▸), *SHELXS97* (Sheldrick, 2008 ▸), *SHELXL2016/6* (Sheldrick, 2015 ▸), *DIAMOND* (Brandenburg, 2005 ▸) and *publCIF* (Westrip, 2010 ▸).

## supplementary crystallographic information

### Caesium dialuminium arsenic(V) hexakis[hydrogen arsenate(V)] (CsAl2AsHAsO46) Crystal data

**Table:**

CsAl_2_As(HAsO_4_)_6_	*D*_x_ = 3.546 Mg m^−^^3^
*M**_r_* = 1101.36	Mo *K*α radiation, λ = 0.71073 Å
Trigonal, *R*3*c*:*H*	Cell parameters from 2513 reflections
*a* = 8.439 (1) Å	θ = 2.4–32.5°
*c* = 50.169 (11) Å	µ = 13.14 mm^−^^1^
*V* = 3094.2 (10) Å^3^	*T* = 293 K
*Z* = 6	Large pseudo-octahedra, colourless
*F*(000) = 3060	0.08 × 0.07 × 0.06 mm

### Caesium dialuminium arsenic(V) hexakis[hydrogen arsenate(V)] (CsAl2AsHAsO46) Data collection

**Table:**

Nonius KappaCCD single-crystal four-circle diffractometer	1153 reflections with *I* > 2σ(*I*)
Radiation source: fine-focus sealed tube	*R*_int_ = 0.014
φ and ω scans	θ_max_ = 32.5°, θ_min_ = 2.4°
Absorption correction: multi-scan (HKL SCALEPACK; Otwinowski *et al.*, 2003)	*h* = −12→12
*T*_min_ = 0.420, *T*_max_ = 0.506	*k* = −10→10
4584 measured reflections	*l* = −75→75
1259 independent reflections	

### Caesium dialuminium arsenic(V) hexakis[hydrogen arsenate(V)] (CsAl2AsHAsO46) Refinement

**Table:**

Refinement on *F*^2^	Secondary atom site location: difference Fourier map
Least-squares matrix: full	Hydrogen site location: difference Fourier map
*R*[*F*^2^ > 2σ(*F*^2^)] = 0.015	All H-atom parameters refined
*wR*(*F*^2^) = 0.034	*w* = 1/[σ^2^(*F*_o_^2^) + (0.0121*P*)^2^ + 14.2108*P*] where *P* = (*F*_o_^2^ + 2*F*_c_^2^)/3
*S* = 1.11	(Δ/σ)_max_ = 0.007
1259 reflections	Δρ_max_ = 0.61 e Å^−^^3^
65 parameters	Δρ_min_ = −0.45 e Å^−^^3^
2 restraints	Extinction correction: SHELXL-2016/6 (Sheldrick 2015), Fc^*^=kFc[1+0.001xFc^2^λ^3^/sin(2θ)]^-1/4^
Primary atom site location: structure-invariant direct methods	Extinction coefficient: 0.000152 (15)

### Caesium dialuminium arsenic(V) hexakis[hydrogen arsenate(V)] (CsAl2AsHAsO46) Special details

**Table:**

Geometry. All esds (except the esd in the dihedral angle between two l.s. planes) are estimated using the full covariance matrix. The cell esds are taken into account individually in the estimation of esds in distances, angles and torsion angles; correlations between esds in cell parameters are only used when they are defined by crystal symmetry. An approximate (isotropic) treatment of cell esds is used for estimating esds involving l.s. planes.

### Caesium dialuminium arsenic(V) hexakis[hydrogen arsenate(V)] (CsAl2AsHAsO46) Fractional atomic coordinates and isotropic or equivalent isotropic displacement parameters (Å^2^)

**Table:**

	*x*	*y*	*z*	*U*_iso_*/*U*_eq_	Occ. (<1)
Cs1A	0.000000	0.000000	0.750000	0.0317 (19)	0.624 (3)
Cs1B	0.000000	−0.018 (3)	0.750000	0.023 (2)	0.1254 (9)
Al1	0.333333	0.666667	0.75637 (2)	0.00616 (14)	
As2	0.333333	0.666667	0.666667	0.00670 (8)	
As1	−0.44835 (2)	−0.41203 (2)	0.71182 (2)	0.00739 (5)	
O1	0.39564 (16)	−0.47264 (16)	0.68660 (2)	0.0100 (2)	
O2	−0.46035 (16)	−0.27705 (16)	0.73495 (2)	0.0099 (2)	
O3	−0.23352 (18)	−0.28644 (19)	0.69863 (3)	0.0178 (3)	
O4	0.48577 (16)	−0.13040 (16)	0.77831 (2)	0.0102 (2)	
H	−0.184 (5)	−0.355 (5)	0.7004 (7)	0.066 (11)*	

### Caesium dialuminium arsenic(V) hexakis[hydrogen arsenate(V)] (CsAl2AsHAsO46) Atomic displacement parameters (Å^2^)

**Table:**

	*U*^11^	*U*^22^	*U*^33^	*U*^12^	*U*^13^	*U*^23^
Cs1A	0.029 (2)	0.029 (2)	0.0366 (19)	0.0146 (12)	0.000	0.000
Cs1B	0.016 (3)	0.028 (4)	0.021 (2)	0.0082 (15)	0.0021 (18)	0.0011 (9)
Al1	0.0066 (2)	0.0066 (2)	0.0053 (3)	0.00329 (11)	0.000	0.000
As2	0.00756 (11)	0.00756 (11)	0.00499 (15)	0.00378 (5)	0.000	0.000
As1	0.00831 (8)	0.00780 (8)	0.00710 (8)	0.00481 (6)	−0.00008 (5)	0.00030 (5)
O1	0.0132 (5)	0.0097 (5)	0.0085 (5)	0.0067 (4)	−0.0030 (4)	0.0000 (4)
O2	0.0106 (5)	0.0099 (5)	0.0097 (5)	0.0055 (4)	0.0007 (4)	−0.0017 (4)
O3	0.0104 (5)	0.0177 (6)	0.0245 (6)	0.0065 (5)	0.0071 (5)	0.0057 (5)
O4	0.0115 (5)	0.0082 (5)	0.0123 (5)	0.0060 (4)	−0.0028 (4)	−0.0035 (4)

### Caesium dialuminium arsenic(V) hexakis[hydrogen arsenate(V)] (CsAl2AsHAsO46) Geometric parameters (Å, º)

**Table:**

Cs1A—Cs1B^i^	0.15 (2)	Cs1B—O2^iii^	3.457 (2)
Cs1A—Cs1B^ii^	0.15 (2)	Cs1B—O3^ii^	3.507 (14)
Cs1A—O3^iii^	3.4066 (15)	Cs1B—O3^v^	3.507 (14)
Cs1A—O3^ii^	3.4066 (16)	Cs1B—O2^v^	3.609 (19)
Cs1A—O3^iv^	3.4066 (16)	Cs1B—O2^ii^	3.609 (19)
Cs1A—O3	3.4066 (16)	Cs1B—O4^vi^	4.018 (19)
Cs1A—O3^v^	3.4066 (15)	Cs1B—O4^vii^	4.018 (19)
Cs1A—O3^i^	3.4066 (16)	Cs1B—As1^i^	4.039 (10)
Cs1A—O2	3.4707 (13)	Cs1B—As1^iv^	4.039 (10)
Cs1A—O2^v^	3.4707 (12)	Cs1B—As1	4.057 (7)
Cs1A—O2^i^	3.4707 (12)	Cs1B—As1^iii^	4.057 (7)
Cs1A—O2^iv^	3.4708 (13)	Al1—O2^viii^	1.8933 (12)
Cs1A—O2^iii^	3.4708 (13)	Al1—O2^ii^	1.8933 (13)
Cs1A—O2^ii^	3.4708 (13)	Al1—O2^ix^	1.8934 (12)
Cs1A—As1	4.1132 (5)	Al1—O4^x^	1.8963 (13)
Cs1A—As1^v^	4.1132 (4)	Al1—O4^i^	1.8963 (12)
Cs1A—As1^i^	4.1132 (5)	Al1—O4^xi^	1.8963 (12)
Cs1A—As1^iv^	4.1133 (4)	As2—O1^xii^	1.8103 (12)
Cs1A—As1^iii^	4.1133 (4)	As2—O1^xiii^	1.8103 (12)
Cs1A—As1^ii^	4.1133 (5)	As2—O1^xi^	1.8104 (11)
Cs1B—Cs1B^i^	0.27 (4)	As2—O1^i^	1.8104 (12)
Cs1B—Cs1B^ii^	0.27 (4)	As2—O1^xiv^	1.8104 (11)
Cs1B—O3^iii^	3.345 (8)	As2—O1^x^	1.8104 (11)
Cs1B—O3	3.345 (8)	As1—O4^iii^	1.6639 (12)
Cs1B—O2^i^	3.352 (16)	As1—O2	1.6641 (11)
Cs1B—O2^iv^	3.352 (16)	As1—O1^xv^	1.7094 (12)
Cs1B—O3^iv^	3.376 (4)	As1—O3	1.7108 (13)
Cs1B—O3^i^	3.376 (4)	O3—H	0.875 (19)
Cs1B—O2	3.457 (2)		
			
Cs1B^i^—Cs1A—Cs1B^ii^	120.0 (2)	O3^v^—Cs1B—O4^vii^	153.1 (3)
Cs1B^i^—Cs1A—O3^iii^	77.07 (3)	O2^v^—Cs1B—O4^vii^	143.57 (3)
Cs1B^ii^—Cs1A—O3^iii^	130.09 (4)	O2^ii^—Cs1B—O4^vii^	146.38 (3)
Cs1B^i^—Cs1A—O3^ii^	77.07 (3)	O4^vi^—Cs1B—O4^vii^	50.4 (3)
Cs1B^ii^—Cs1A—O3^ii^	65.14 (12)	Cs1B^i^—Cs1B—As1^i^	91.9 (2)
O3^iii^—Cs1A—O3^ii^	154.15 (5)	Cs1B^ii^—Cs1B—As1^i^	141.11 (14)
Cs1B^i^—Cs1A—O3^iv^	130.09 (3)	O3^iii^—Cs1B—As1^i^	78.8 (2)
Cs1B^ii^—Cs1A—O3^iv^	65.14 (12)	O3—Cs1B—As1^i^	80.6 (2)
O3^iii^—Cs1A—O3^iv^	68.99 (4)	O2^i^—Cs1B—As1^i^	23.77 (5)
O3^ii^—Cs1A—O3^iv^	130.29 (5)	O2^iv^—Cs1B—As1^i^	101.5 (5)
Cs1B^i^—Cs1A—O3	130.09 (3)	O3^iv^—Cs1B—As1^i^	137.7 (6)
Cs1B^ii^—Cs1A—O3	77.07 (14)	O3^i^—Cs1B—As1^i^	24.66 (9)
O3^iii^—Cs1A—O3	130.29 (5)	O2—Cs1B—As1^i^	126.1 (3)
O3^ii^—Cs1A—O3	68.99 (4)	O2^iii^—Cs1B—As1^i^	49.09 (10)
O3^iv^—Cs1A—O3	99.81 (5)	O3^ii^—Cs1B—As1^i^	92.71 (5)
Cs1B^i^—Cs1A—O3^v^	65.14 (4)	O3^v^—Cs1B—As1^i^	125.43 (12)
Cs1B^ii^—Cs1A—O3^v^	77.07 (14)	O2^v^—Cs1B—As1^i^	143.1 (4)
O3^iii^—Cs1A—O3^v^	68.99 (4)	O2^ii^—Cs1B—As1^i^	92.74 (12)
O3^ii^—Cs1A—O3^v^	99.81 (5)	O4^vi^—Cs1B—As1^i^	64.3 (3)
O3^iv^—Cs1A—O3^v^	68.99 (4)	O4^vii^—Cs1B—As1^i^	65.5 (3)
O3—Cs1A—O3^v^	154.15 (5)	Cs1B^i^—Cs1B—As1^iv^	141.1 (3)
Cs1B^i^—Cs1A—O3^i^	65.14 (4)	Cs1B^ii^—Cs1B—As1^iv^	91.9 (4)
Cs1B^ii^—Cs1A—O3^i^	130.09 (4)	O3^iii^—Cs1B—As1^iv^	80.6 (2)
O3^iii^—Cs1A—O3^i^	99.81 (5)	O3—Cs1B—As1^iv^	78.8 (2)
O3^ii^—Cs1A—O3^i^	68.99 (4)	O2^i^—Cs1B—As1^iv^	101.5 (5)
O3^iv^—Cs1A—O3^i^	154.15 (5)	O2^iv^—Cs1B—As1^iv^	23.77 (5)
O3—Cs1A—O3^i^	68.99 (4)	O3^iv^—Cs1B—As1^iv^	24.66 (9)
O3^v^—Cs1A—O3^i^	130.29 (5)	O3^i^—Cs1B—As1^iv^	137.7 (6)
Cs1B^i^—Cs1A—O2	153.70 (4)	O2—Cs1B—As1^iv^	49.09 (10)
Cs1B^ii^—Cs1A—O2	38.5 (2)	O2^iii^—Cs1B—As1^iv^	126.1 (3)
O3^iii^—Cs1A—O2	127.04 (3)	O3^ii^—Cs1B—As1^iv^	125.43 (12)
O3^ii^—Cs1A—O2	78.39 (3)	O3^v^—Cs1B—As1^iv^	92.71 (5)
O3^iv^—Cs1A—O2	62.83 (3)	O2^v^—Cs1B—As1^iv^	92.74 (12)
O3—Cs1A—O2	45.72 (3)	O2^ii^—Cs1B—As1^iv^	143.1 (4)
O3^v^—Cs1A—O2	110.37 (3)	O4^vi^—Cs1B—As1^iv^	65.5 (3)
O3^i^—Cs1A—O2	113.93 (3)	O4^vii^—Cs1B—As1^iv^	64.3 (3)
Cs1B^i^—Cs1A—O2^v^	83.46 (2)	As1^i^—Cs1B—As1^iv^	124.1 (5)
Cs1B^ii^—Cs1A—O2^v^	38.5 (2)	Cs1B^i^—Cs1B—As1	135.2 (3)
O3^iii^—Cs1A—O2^v^	113.93 (3)	Cs1B^ii^—Cs1B—As1	84.4 (4)
O3^ii^—Cs1A—O2^v^	62.83 (3)	O3^iii^—Cs1B—As1	129.9 (5)
O3^iv^—Cs1A—O2^v^	78.39 (3)	O3—Cs1B—As1	24.38 (5)
O3—Cs1A—O2^v^	110.37 (3)	O2^i^—Cs1B—As1	96.4 (4)
O3^v^—Cs1A—O2^v^	45.72 (3)	O2^iv^—Cs1B—As1	49.35 (14)
O3^i^—Cs1A—O2^v^	127.04 (3)	O3^iv^—Cs1B—As1	78.22 (15)
O2—Cs1A—O2^v^	77.02 (4)	O3^i^—Cs1B—As1	94.4 (2)
Cs1B^i^—Cs1A—O2^i^	83.46 (2)	O2—Cs1B—As1	23.92 (7)
Cs1B^ii^—Cs1A—O2^i^	153.70 (19)	O2^iii^—Cs1B—As1	149.8 (5)
O3^iii^—Cs1A—O2^i^	62.83 (3)	O3^ii^—Cs1B—As1	78.48 (6)
O3^ii^—Cs1A—O2^i^	113.93 (3)	O3^v^—Cs1B—As1	132.2 (2)
O3^iv^—Cs1A—O2^i^	110.37 (3)	O2^v^—Cs1B—As1	96.79 (18)
O3—Cs1A—O2^i^	78.39 (3)	O2^ii^—Cs1B—As1	121.4 (3)
O3^v^—Cs1A—O2^i^	127.04 (3)	O4^vi^—Cs1B—As1	92.2 (4)
O3^i^—Cs1A—O2^i^	45.72 (3)	O4^vii^—Cs1B—As1	47.32 (16)
O2—Cs1A—O2^i^	115.404 (13)	As1^i^—Cs1B—As1	102.3 (3)
O2^v^—Cs1A—O2^i^	166.91 (4)	As1^iv^—Cs1B—As1	57.07 (13)
Cs1B^i^—Cs1A—O2^iv^	153.70 (4)	Cs1B^i^—Cs1B—As1^iii^	84.4 (2)
Cs1B^ii^—Cs1A—O2^iv^	83.5 (2)	Cs1B^ii^—Cs1B—As1^iii^	135.20 (15)
O3^iii^—Cs1A—O2^iv^	78.39 (3)	O3^iii^—Cs1B—As1^iii^	24.38 (5)
O3^ii^—Cs1A—O2^iv^	127.04 (3)	O3—Cs1B—As1^iii^	129.9 (5)
O3^iv^—Cs1A—O2^iv^	45.72 (3)	O2^i^—Cs1B—As1^iii^	49.35 (14)
O3—Cs1A—O2^iv^	62.83 (3)	O2^iv^—Cs1B—As1^iii^	96.4 (4)
O3^v^—Cs1A—O2^iv^	113.93 (3)	O3^iv^—Cs1B—As1^iii^	94.4 (2)
O3^i^—Cs1A—O2^iv^	110.37 (3)	O3^i^—Cs1B—As1^iii^	78.22 (15)
O2—Cs1A—O2^iv^	52.60 (4)	O2—Cs1B—As1^iii^	149.8 (5)
O2^v^—Cs1A—O2^iv^	115.404 (13)	O2^iii^—Cs1B—As1^iii^	23.92 (7)
O2^i^—Cs1A—O2^iv^	77.02 (4)	O3^ii^—Cs1B—As1^iii^	132.2 (2)
Cs1B^i^—Cs1A—O2^iii^	38.51 (2)	O3^v^—Cs1B—As1^iii^	78.48 (6)
Cs1B^ii^—Cs1A—O2^iii^	153.70 (19)	O2^v^—Cs1B—As1^iii^	121.4 (3)
O3^iii^—Cs1A—O2^iii^	45.72 (3)	O2^ii^—Cs1B—As1^iii^	96.80 (18)
O3^ii^—Cs1A—O2^iii^	110.37 (3)	O4^vi^—Cs1B—As1^iii^	47.32 (16)
O3^iv^—Cs1A—O2^iii^	113.93 (3)	O4^vii^—Cs1B—As1^iii^	92.2 (4)
O3—Cs1A—O2^iii^	127.04 (3)	As1^i^—Cs1B—As1^iii^	57.07 (13)
O3^v^—Cs1A—O2^iii^	78.39 (3)	As1^iv^—Cs1B—As1^iii^	102.3 (3)
O3^i^—Cs1A—O2^iii^	62.83 (3)	As1—Cs1B—As1^iii^	138.7 (6)
O2—Cs1A—O2^iii^	166.91 (4)	O2^viii^—Al1—O2^ii^	90.96 (6)
O2^v^—Cs1A—O2^iii^	115.403 (13)	O2^viii^—Al1—O2^ix^	90.96 (6)
O2^i^—Cs1A—O2^iii^	52.60 (4)	O2^ii^—Al1—O2^ix^	90.96 (6)
O2^iv^—Cs1A—O2^iii^	115.402 (13)	O2^viii^—Al1—O4^x^	88.82 (5)
Cs1B^i^—Cs1A—O2^ii^	38.51 (2)	O2^ii^—Al1—O4^x^	178.50 (6)
Cs1B^ii^—Cs1A—O2^ii^	83.5 (2)	O2^ix^—Al1—O4^x^	90.53 (5)
O3^iii^—Cs1A—O2^ii^	110.37 (3)	O2^viii^—Al1—O4^i^	178.50 (6)
O3^ii^—Cs1A—O2^ii^	45.72 (3)	O2^ii^—Al1—O4^i^	90.53 (5)
O3^iv^—Cs1A—O2^ii^	127.04 (3)	O2^ix^—Al1—O4^i^	88.82 (5)
O3—Cs1A—O2^ii^	113.93 (3)	O4^x^—Al1—O4^i^	89.70 (6)
O3^v^—Cs1A—O2^ii^	62.83 (3)	O2^viii^—Al1—O4^xi^	90.53 (5)
O3^i^—Cs1A—O2^ii^	78.39 (3)	O2^ii^—Al1—O4^xi^	88.82 (5)
O2—Cs1A—O2^ii^	115.404 (13)	O2^ix^—Al1—O4^xi^	178.50 (6)
O2^v^—Cs1A—O2^ii^	52.60 (4)	O4^x^—Al1—O4^xi^	89.70 (6)
O2^i^—Cs1A—O2^ii^	115.404 (13)	O4^i^—Al1—O4^xi^	89.70 (6)
O2^iv^—Cs1A—O2^ii^	166.91 (4)	O2^viii^—Al1—Cs1B^x^	123.30 (10)
O2^iii^—Cs1A—O2^ii^	77.02 (4)	O2^ii^—Al1—Cs1B^x^	99.54 (13)
Cs1B^i^—Cs1A—As1	151.960 (16)	O2^ix^—Al1—Cs1B^x^	33.98 (6)
Cs1B^ii^—Cs1A—As1	60.19 (18)	O4^x^—Al1—Cs1B^x^	81.81 (12)
O3^iii^—Cs1A—As1	126.11 (2)	O4^i^—Al1—Cs1B^x^	56.21 (9)
O3^ii^—Cs1A—As1	78.79 (2)	O4^xi^—Al1—Cs1B^x^	144.65 (7)
O3^iv^—Cs1A—As1	77.08 (3)	O2^viii^—Al1—Cs1B^i^	99.54 (12)
O3—Cs1A—As1	24.02 (2)	O2^ii^—Al1—Cs1B^i^	33.98 (5)
O3^v^—Cs1A—As1	133.70 (2)	O2^ix^—Al1—Cs1B^i^	123.30 (9)
O3^i^—Cs1A—As1	92.93 (3)	O4^x^—Al1—Cs1B^i^	144.65 (6)
O2—Cs1A—As1	23.442 (18)	O4^i^—Al1—Cs1B^i^	81.81 (12)
O2^v^—Cs1A—As1	98.010 (19)	O4^xi^—Al1—Cs1B^i^	56.21 (9)
O2^i^—Cs1A—As1	93.54 (2)	Cs1B^x^—Al1—Cs1B^i^	119.552 (13)
O2^iv^—Cs1A—As1	48.32 (2)	O2^viii^—Al1—Cs1B^xi^	33.98 (6)
O2^iii^—Cs1A—As1	146.060 (19)	O2^ii^—Al1—Cs1B^xi^	123.30 (10)
O2^ii^—Cs1A—As1	123.48 (2)	O2^ix^—Al1—Cs1B^xi^	99.54 (14)
Cs1B^i^—Cs1A—As1^v^	67.330 (11)	O4^x^—Al1—Cs1B^xi^	56.21 (10)
Cs1B^ii^—Cs1A—As1^v^	60.19 (18)	O4^i^—Al1—Cs1B^xi^	144.65 (7)
O3^iii^—Cs1A—As1^v^	92.93 (3)	O4^xi^—Al1—Cs1B^xi^	81.81 (14)
O3^ii^—Cs1A—As1^v^	77.09 (3)	Cs1B^x^—Al1—Cs1B^xi^	119.552 (7)
O3^iv^—Cs1A—As1^v^	78.78 (2)	Cs1B^i^—Al1—Cs1B^xi^	119.552 (4)
O3—Cs1A—As1^v^	133.71 (2)	O2^viii^—Al1—Cs1A^x^	122.73 (4)
O3^v^—Cs1A—As1^v^	24.02 (2)	O2^ii^—Al1—Cs1A^x^	100.35 (4)
O3^i^—Cs1A—As1^v^	126.11 (2)	O2^ix^—Al1—Cs1A^x^	33.72 (4)
O2—Cs1A—As1^v^	98.010 (19)	O4^x^—Al1—Cs1A^x^	81.01 (3)
O2^v^—Cs1A—As1^v^	23.441 (19)	O4^i^—Al1—Cs1A^x^	56.75 (4)
O2^i^—Cs1A—As1^v^	146.06 (2)	O4^xi^—Al1—Cs1A^x^	144.92 (4)
O2^iv^—Cs1A—As1^v^	123.484 (19)	Cs1B^x^—Al1—Cs1A^x^	0.93 (14)
O2^iii^—Cs1A—As1^v^	93.54 (2)	Cs1B^i^—Al1—Cs1A^x^	120.48 (13)
O2^ii^—Cs1A—As1^v^	48.32 (2)	Cs1B^xi^—Al1—Cs1A^x^	118.65 (14)
As1—Cs1A—As1^v^	120.371 (6)	O2^viii^—Al1—Cs1A	100.35 (4)
Cs1B^i^—Cs1A—As1^i^	67.330 (8)	O2^ii^—Al1—Cs1A	33.72 (4)
Cs1B^ii^—Cs1A—As1^i^	151.96 (3)	O2^ix^—Al1—Cs1A	122.73 (4)
O3^iii^—Cs1A—As1^i^	77.09 (3)	O4^x^—Al1—Cs1A	144.92 (4)
O3^ii^—Cs1A—As1^i^	92.93 (3)	O4^i^—Al1—Cs1A	81.01 (4)
O3^iv^—Cs1A—As1^i^	133.71 (2)	O4^xi^—Al1—Cs1A	56.75 (4)
O3—Cs1A—As1^i^	78.79 (2)	Cs1B^x^—Al1—Cs1A	118.65 (14)
O3^v^—Cs1A—As1^i^	126.11 (2)	Cs1B^i^—Al1—Cs1A	0.93 (13)
O3^i^—Cs1A—As1^i^	24.02 (2)	Cs1B^xi^—Al1—Cs1A	120.48 (13)
O2—Cs1A—As1^i^	123.48 (2)	Cs1A^x^—Al1—Cs1A	119.576 (2)
O2^v^—Cs1A—As1^i^	146.06 (2)	O2^viii^—Al1—Cs1A^viii^	33.72 (4)
O2^i^—Cs1A—As1^i^	23.441 (19)	O2^ii^—Al1—Cs1A^viii^	122.74 (4)
O2^iv^—Cs1A—As1^i^	98.010 (19)	O2^ix^—Al1—Cs1A^viii^	100.35 (4)
O2^iii^—Cs1A—As1^i^	48.32 (2)	O4^x^—Al1—Cs1A^viii^	56.75 (4)
O2^ii^—Cs1A—As1^i^	93.54 (2)	O4^i^—Al1—Cs1A^viii^	144.92 (4)
As1—Cs1A—As1^i^	100.065 (9)	O4^xi^—Al1—Cs1A^viii^	81.01 (4)
As1^v^—Cs1A—As1^i^	134.660 (5)	Cs1B^x^—Al1—Cs1A^viii^	120.48 (14)
Cs1B^i^—Cs1A—As1^iv^	151.959 (16)	Cs1B^i^—Al1—Cs1A^viii^	118.65 (13)
Cs1B^ii^—Cs1A—As1^iv^	67.33 (18)	Cs1B^xi^—Al1—Cs1A^viii^	0.93 (14)
O3^iii^—Cs1A—As1^iv^	78.79 (2)	Cs1A^x^—Al1—Cs1A^viii^	119.576 (2)
O3^ii^—Cs1A—As1^iv^	126.11 (2)	Cs1A—Al1—Cs1A^viii^	119.576 (2)
O3^iv^—Cs1A—As1^iv^	24.02 (2)	O1^xii^—As2—O1^xiii^	92.41 (5)
O3—Cs1A—As1^iv^	77.08 (3)	O1^xii^—As2—O1^xi^	180.0
O3^v^—Cs1A—As1^iv^	92.93 (3)	O1^xiii^—As2—O1^xi^	87.59 (5)
O3^i^—Cs1A—As1^iv^	133.70 (2)	O1^xii^—As2—O1^i^	87.59 (5)
O2—Cs1A—As1^iv^	48.31 (2)	O1^xiii^—As2—O1^i^	180.00 (10)
O2^v^—Cs1A—As1^iv^	93.54 (2)	O1^xi^—As2—O1^i^	92.41 (5)
O2^i^—Cs1A—As1^iv^	98.010 (19)	O1^xii^—As2—O1^xiv^	92.41 (5)
O2^iv^—Cs1A—As1^iv^	23.441 (18)	O1^xiii^—As2—O1^xiv^	92.41 (5)
O2^iii^—Cs1A—As1^iv^	123.482 (19)	O1^xi^—As2—O1^xiv^	87.59 (5)
O2^ii^—Cs1A—As1^iv^	146.060 (19)	O1^i^—As2—O1^xiv^	87.59 (5)
As1—Cs1A—As1^iv^	56.080 (13)	O1^xii^—As2—O1^x^	87.59 (5)
As1^v^—Cs1A—As1^iv^	100.065 (7)	O1^xiii^—As2—O1^x^	87.59 (5)
As1^i^—Cs1A—As1^iv^	120.371 (6)	O1^xi^—As2—O1^x^	92.40 (5)
Cs1B^i^—Cs1A—As1^iii^	60.186 (15)	O1^i^—As2—O1^x^	92.40 (5)
Cs1B^ii^—Cs1A—As1^iii^	151.96 (3)	O1^xiv^—As2—O1^x^	180.00 (7)
O3^iii^—Cs1A—As1^iii^	24.02 (2)	O4^iii^—As1—O2	117.14 (6)
O3^ii^—Cs1A—As1^iii^	133.70 (2)	O4^iii^—As1—O1^xv^	101.04 (6)
O3^iv^—Cs1A—As1^iii^	92.93 (3)	O2—As1—O1^xv^	114.80 (6)
O3—Cs1A—As1^iii^	126.11 (2)	O4^iii^—As1—O3	110.50 (6)
O3^v^—Cs1A—As1^iii^	78.79 (2)	O2—As1—O3	104.71 (7)
O3^i^—Cs1A—As1^iii^	77.08 (3)	O1^xv^—As1—O3	108.55 (7)
O2—Cs1A—As1^iii^	146.058 (19)	O4^iii^—As1—Cs1B^ii^	111.95 (18)
O2^v^—Cs1A—As1^iii^	123.483 (19)	O2—As1—Cs1B^ii^	54.3 (3)
O2^i^—Cs1A—As1^iii^	48.32 (2)	O1^xv^—As1—Cs1B^ii^	146.59 (15)
O2^iv^—Cs1A—As1^iii^	93.54 (2)	O3—As1—Cs1B^ii^	55.4 (2)
O2^iii^—Cs1A—As1^iii^	23.441 (19)	O4^iii^—As1—Cs1B	108.9 (3)
O2^ii^—Cs1A—As1^iii^	98.011 (19)	O2—As1—Cs1B	57.4 (2)
As1—Cs1A—As1^iii^	134.660 (5)	O1^xv^—As1—Cs1B	149.1 (2)
As1^v^—Cs1A—As1^iii^	100.065 (7)	O3—As1—Cs1B	53.82 (8)
As1^i^—Cs1A—As1^iii^	56.081 (12)	Cs1B^ii^—As1—Cs1B	3.7 (5)
As1^iv^—Cs1A—As1^iii^	100.064 (7)	O4^iii^—As1—Cs1A	110.78 (4)
Cs1B^i^—Cs1A—As1^ii^	60.186 (14)	O2—As1—Cs1A	56.07 (4)
Cs1B^ii^—Cs1A—As1^ii^	67.33 (18)	O1^xv^—As1—Cs1A	147.44 (4)
O3^iii^—Cs1A—As1^ii^	133.70 (2)	O3—As1—Cs1A	54.16 (5)
O3^ii^—Cs1A—As1^ii^	24.02 (2)	Cs1B^ii^—As1—Cs1A	1.9 (3)
O3^iv^—Cs1A—As1^ii^	126.11 (2)	Cs1B—As1—Cs1A	2.0 (3)
O3—Cs1A—As1^ii^	92.93 (3)	O4^iii^—As1—Cs1B^i^	111.44 (10)
O3^v^—Cs1A—As1^ii^	77.08 (3)	O2—As1—Cs1B^i^	56.52 (7)
O3^i^—Cs1A—As1^ii^	78.79 (2)	O1^xv^—As1—Cs1B^i^	146.64 (11)
O2—Cs1A—As1^ii^	93.55 (2)	O3—As1—Cs1B^i^	53.34 (12)
O2^v^—Cs1A—As1^ii^	48.32 (2)	Cs1B^ii^—As1—Cs1B^i^	2.2 (3)
O2^i^—Cs1A—As1^ii^	123.48 (2)	Cs1B—As1—Cs1B^i^	2.5 (4)
O2^iv^—Cs1A—As1^ii^	146.060 (19)	Cs1A—As1—Cs1B^i^	0.97 (13)
O2^iii^—Cs1A—As1^ii^	98.011 (19)	O4^iii^—As1—Cs1B^xvi^	44.17 (5)
O2^ii^—Cs1A—As1^ii^	23.441 (19)	O2—As1—Cs1B^xvi^	93.27 (8)
As1—Cs1A—As1^ii^	100.065 (9)	O1^xv^—As1—Cs1B^xvi^	78.98 (10)
As1^v^—Cs1A—As1^ii^	56.081 (13)	O3—As1—Cs1B^xvi^	154.47 (5)
As1^i^—Cs1A—As1^ii^	100.065 (9)	Cs1B^ii^—As1—Cs1B^xvi^	129.10 (15)
As1^iv^—Cs1A—As1^ii^	134.660 (5)	Cs1B—As1—Cs1B^xvi^	128.6 (2)
As1^iii^—Cs1A—As1^ii^	120.371 (6)	Cs1A—As1—Cs1B^xvi^	129.41 (10)
Cs1B^i^—Cs1B—Cs1B^ii^	60.00 (4)	Cs1B^i^—As1—Cs1B^xvi^	130.38 (4)
Cs1B^i^—Cs1B—O3^iii^	94.4 (3)	O4^iii^—As1—Cs1A^xvii^	44.29 (4)
Cs1B^ii^—Cs1B—O3^iii^	125.9 (2)	O2—As1—Cs1A^xvii^	93.71 (4)
Cs1B^i^—Cs1B—O3	125.9 (3)	O1^xv^—As1—Cs1A^xvii^	78.36 (4)
Cs1B^ii^—Cs1B—O3	94.4 (4)	O3—As1—Cs1A^xvii^	154.48 (5)
O3^iii^—Cs1B—O3	135.1 (7)	Cs1B^ii^—As1—Cs1A^xvii^	129.75 (5)
Cs1B^i^—Cs1B—O2^i^	111.1 (2)	Cs1B—As1—Cs1A^xvii^	129.29 (11)
Cs1B^ii^—Cs1B—O2^i^	164.59 (9)	Cs1A—As1—Cs1A^xvii^	130.055 (13)
O3^iii^—Cs1B—O2^i^	64.7 (3)	Cs1B^i^—As1—Cs1A^xvii^	131.03 (14)
O3—Cs1B—O2^i^	80.9 (4)	Cs1B^xvi^—As1—Cs1A^xvii^	0.65 (10)
Cs1B^i^—Cs1B—O2^iv^	164.6 (2)	O4^iii^—As1—Cs1B^xviii^	45.8 (2)
Cs1B^ii^—Cs1B—O2^iv^	111.1 (4)	O2—As1—Cs1B^xviii^	92.99 (12)
O3^iii^—Cs1B—O2^iv^	80.9 (4)	O1^xv^—As1—Cs1B^xviii^	77.45 (15)
O3—Cs1B—O2^iv^	64.7 (3)	O3—As1—Cs1B^xviii^	155.9 (2)
O2^i^—Cs1B—O2^iv^	80.3 (5)	Cs1B^ii^—As1—Cs1B^xviii^	130.167 (13)
Cs1B^i^—Cs1B—O3^iv^	117.8 (3)	Cs1B—As1—Cs1B^xviii^	129.84 (3)
Cs1B^ii^—Cs1B—O3^iv^	81.1 (4)	Cs1A—As1—Cs1B^xviii^	130.54 (7)
O3^iii^—Cs1B—O3^iv^	70.08 (15)	Cs1B^i^—As1—Cs1B^xviii^	131.5 (2)
O3—Cs1B—O3^iv^	101.7 (3)	Cs1B^xvi^—As1—Cs1B^xviii^	1.8 (3)
O2^i^—Cs1B—O3^iv^	114.1 (5)	Cs1A^xvii^—As1—Cs1B^xviii^	1.5 (2)
O2^iv^—Cs1B—O3^iv^	46.81 (15)	O4^iii^—As1—Cs1B^xvii^	42.9 (2)
Cs1B^i^—Cs1B—O3^i^	81.1 (3)	O2—As1—Cs1B^xvii^	94.85 (18)
Cs1B^ii^—Cs1B—O3^i^	117.8 (2)	O1^xv^—As1—Cs1B^xvii^	78.69 (8)
O3^iii^—Cs1B—O3^i^	101.7 (3)	O3—As1—Cs1B^xvii^	153.1 (2)
O3—Cs1B—O3^i^	70.08 (15)	Cs1B^ii^—As1—Cs1B^xvii^	129.92 (2)
O2^i^—Cs1B—O3^i^	46.81 (15)	Cs1B—As1—Cs1B^xvii^	129.35 (12)
O2^iv^—Cs1B—O3^i^	114.1 (5)	Cs1A—As1—Cs1B^xvii^	130.167 (19)
O3^iv^—Cs1B—O3^i^	159.2 (7)	Cs1B^i^—As1—Cs1B^xvii^	131.14 (15)
Cs1B^i^—Cs1B—O2	123.2 (4)	Cs1B^xvi^—As1—Cs1B^xvii^	1.6 (2)
Cs1B^ii^—Cs1B—O2	64.8 (5)	Cs1A^xvii^—As1—Cs1B^xvii^	1.4 (2)
O3^iii^—Cs1B—O2	129.6 (3)	Cs1B^xviii^—As1—Cs1B^xvii^	2.9 (4)
O3—Cs1B—O2	46.24 (7)	As1^xix^—O1—As2^xx^	130.17 (7)
O2^i^—Cs1B—O2	119.0 (5)	As1^xix^—O1—Cs1B^vi^	81.42 (16)
O2^iv^—Cs1B—O2	53.68 (15)	As2^xx^—O1—Cs1B^vi^	129.94 (11)
O3^iv^—Cs1B—O2	63.29 (6)	As1^xix^—O1—Cs1A^xx^	82.55 (4)
O3^i^—Cs1B—O2	115.10 (14)	As2^xx^—O1—Cs1A^xx^	129.26 (5)
Cs1B^i^—Cs1B—O2^iii^	64.8 (3)	Cs1B^vi^—O1—Cs1A^xx^	1.14 (17)
Cs1B^ii^—Cs1B—O2^iii^	123.2 (2)	As1^xix^—O1—Cs1B^xxi^	83.66 (17)
O3^iii^—Cs1B—O2^iii^	46.23 (7)	As2^xx^—O1—Cs1B^xxi^	127.6 (2)
O3—Cs1B—O2^iii^	129.7 (3)	Cs1B^vi^—O1—Cs1B^xxi^	2.5 (3)
O2^i^—Cs1B—O2^iii^	53.68 (15)	Cs1A^xx^—O1—Cs1B^xxi^	1.7 (2)
O2^iv^—Cs1B—O2^iii^	119.0 (5)	As1—O2—Al1^xvii^	125.94 (7)
O3^iv^—Cs1B—O2^iii^	115.10 (14)	As1—O2—Cs1B^ii^	101.9 (2)
O3^i^—Cs1B—O2^iii^	63.29 (6)	Al1^xvii^—O2—Cs1B^ii^	127.63 (17)
O2—Cs1B—O2^iii^	172.0 (7)	As1—O2—Cs1B	98.7 (3)
Cs1B^i^—Cs1B—O3^ii^	58.4 (2)	Al1^xvii^—O2—Cs1B	129.54 (14)
Cs1B^ii^—Cs1B—O3^ii^	50.60 (16)	Cs1B^ii^—O2—Cs1B	4.1 (6)
O3^iii^—Cs1B—O3^ii^	151.4 (4)	As1—O2—Cs1A	100.49 (5)
O3—Cs1B—O3^ii^	68.50 (9)	Al1^xvii^—O2—Cs1A	128.67 (5)
O2^i^—Cs1B—O3^ii^	114.37 (6)	Cs1B^ii^—O2—Cs1A	1.6 (2)
O2^iv^—Cs1B—O3^ii^	127.63 (7)	Cs1B—O2—Cs1A	2.5 (4)
O3^iv^—Cs1B—O3^ii^	127.8 (4)	As1—O2—Cs1B^i^	100.86 (7)
O3^i^—Cs1B—O3^ii^	68.17 (13)	Al1^xvii^—O2—Cs1B^i^	128.74 (5)
O2—Cs1B—O3^ii^	77.24 (17)	Cs1B^ii^—O2—Cs1B^i^	1.12 (16)
O2^iii^—Cs1B—O3^ii^	108.3 (3)	Cs1B—O2—Cs1B^i^	3.5 (5)
Cs1B^i^—Cs1B—O3^v^	50.6 (2)	Cs1A—O2—Cs1B^i^	1.08 (14)
Cs1B^ii^—Cs1B—O3^v^	58.37 (14)	As1—O3—Cs1B	101.80 (6)
O3^iii^—Cs1B—O3^v^	68.50 (9)	As1—O3—Cs1B^ii^	99.9 (3)
O3—Cs1B—O3^v^	151.4 (4)	Cs1B—O3—Cs1B^ii^	4.5 (6)
O2^i^—Cs1B—O3^v^	127.63 (7)	As1—O3—Cs1A	101.82 (6)
O2^iv^—Cs1B—O3^v^	114.37 (6)	Cs1B—O3—Cs1A	2.4 (3)
O3^iv^—Cs1B—O3^v^	68.17 (13)	Cs1B^ii^—O3—Cs1A	2.5 (4)
O3^i^—Cs1B—O3^v^	127.8 (4)	As1—O3—Cs1B^i^	103.6 (2)
O2—Cs1B—O3^v^	108.3 (3)	Cs1B—O3—Cs1B^i^	3.5 (5)
O2^iii^—Cs1B—O3^v^	77.24 (17)	Cs1B^ii^—O3—Cs1B^i^	3.8 (5)
O3^ii^—Cs1B—O3^v^	96.0 (5)	Cs1A—O3—Cs1B^i^	1.9 (3)
Cs1B^i^—Cs1B—O2^v^	53.28 (17)	As1—O3—H	105 (3)
Cs1B^ii^—Cs1B—O2^v^	14.29 (7)	Cs1B—O3—H	93 (3)
O3^iii^—Cs1B—O2^v^	112.0 (3)	Cs1B^ii^—O3—H	98 (2)
O3—Cs1B—O2^v^	108.5 (3)	Cs1A—O3—H	96 (2)
O2^i^—Cs1B—O2^v^	164.4 (4)	Cs1B^i^—O3—H	96 (3)
O2^iv^—Cs1B—O2^v^	114.85 (9)	As1^iii^—O4—Al1^xx^	127.34 (7)
O3^iv^—Cs1B—O2^v^	76.9 (2)	As1^iii^—O4—Cs1B^xxii^	119.06 (10)
O3^i^—Cs1B—O2^v^	123.6 (5)	Al1^xx^—O4—Cs1B^xxii^	100.69 (5)
O2—Cs1B—O2^v^	75.4 (2)	As1^iii^—O4—Cs1A^xix^	119.48 (5)
O2^iii^—Cs1B—O2^v^	112.3 (4)	Al1^xx^—O4—Cs1A^xix^	100.82 (4)
O3^ii^—Cs1B—O2^v^	60.5 (3)	Cs1B^xxii^—O4—Cs1A^xix^	0.90 (13)
O3^v^—Cs1B—O2^v^	44.1 (2)	As1^iii^—O4—Cs1B^xix^	121.4 (3)
Cs1B^i^—Cs1B—O2^ii^	14.29 (3)	Al1^xx^—O4—Cs1B^xix^	99.51 (19)
Cs1B^ii^—Cs1B—O2^ii^	53.28 (3)	Cs1B^xxii^—O4—Cs1B^xix^	2.7 (4)
O3^iii^—Cs1B—O2^ii^	108.5 (3)	Cs1A^xix^—O4—Cs1B^xix^	2.0 (3)
O3—Cs1B—O2^ii^	112.0 (3)	As1^iii^—O4—Cs1B^xxiii^	117.9 (2)
O2^i^—Cs1B—O2^ii^	114.85 (9)	Al1^xx^—O4—Cs1B^xxiii^	102.2 (2)
O2^iv^—Cs1B—O2^ii^	164.4 (4)	Cs1B^xxii^—O4—Cs1B^xxiii^	1.5 (2)
O3^iv^—Cs1B—O2^ii^	123.6 (5)	Cs1A^xix^—O4—Cs1B^xxiii^	1.6 (2)
O3^i^—Cs1B—O2^ii^	76.9 (2)	Cs1B^xix^—O4—Cs1B^xxiii^	3.5 (5)
O2—Cs1B—O2^ii^	112.3 (4)	As1^iii^—O4—Cs1B	52.18 (19)
O2^iii^—Cs1B—O2^ii^	75.4 (2)	Al1^xx^—O4—Cs1B	75.5 (2)
O3^ii^—Cs1B—O2^ii^	44.1 (2)	Cs1B^xxii^—O4—Cs1B	135.11 (6)
O3^v^—Cs1B—O2^ii^	60.5 (3)	Cs1A^xix^—O4—Cs1B	136.01 (8)
O2^v^—Cs1B—O2^ii^	50.4 (3)	Cs1B^xix^—O4—Cs1B	137.3 (2)
Cs1B^i^—Cs1B—O4^vi^	131.69 (8)	Cs1B^xxiii^—O4—Cs1B	135.55 (3)
Cs1B^ii^—Cs1B—O4^vi^	154.46 (10)	As1^iii^—O4—Cs1B^i^	49.73 (17)
O3^iii^—Cs1B—O4^vi^	42.3 (2)	Al1^xx^—O4—Cs1B^i^	78.1 (2)
O3—Cs1B—O4^vi^	92.7 (5)	Cs1B^xxii^—O4—Cs1B^i^	133.5 (3)
O2^i^—Cs1B—O4^vi^	40.9 (2)	Cs1A^xix^—O4—Cs1B^i^	134.38 (18)
O2^iv^—Cs1B—O4^vi^	51.1 (3)	Cs1B^xix^—O4—Cs1B^i^	135.76 (3)
O3^iv^—Cs1B—O4^vi^	73.4 (3)	Cs1B^xxiii^—O4—Cs1B^i^	133.8 (3)
O3^i^—Cs1B—O4^vi^	87.7 (4)	Cs1B—O4—Cs1B^i^	3.1 (4)
O2—Cs1B—O4^vi^	104.2 (4)	As1^iii^—O4—Cs1A	50.92 (3)
O2^iii^—Cs1B—O4^vi^	68.2 (2)	Al1^xx^—O4—Cs1A	76.77 (3)
O3^ii^—Cs1B—O4^vi^	153.1 (3)	Cs1B^xxii^—O4—Cs1A	134.61 (13)
O3^v^—Cs1B—O4^vi^	108.65 (13)	Cs1A^xix^—O4—Cs1A	135.51 (3)
O2^v^—Cs1B—O4^vi^	146.38 (3)	Cs1B^xix^—O4—Cs1A	136.84 (18)
O2^ii^—Cs1B—O4^vi^	143.57 (3)	Cs1B^xxiii^—O4—Cs1A	135.01 (8)
Cs1B^i^—Cs1B—O4^vii^	154.46 (3)	Cs1B—O4—Cs1A	1.4 (2)
Cs1B^ii^—Cs1B—O4^vii^	131.7 (2)	Cs1B^i^—O4—Cs1A	1.7 (3)
O3^iii^—Cs1B—O4^vii^	92.7 (5)	As1^iii^—O4—Cs1B^ii^	50.88 (4)
O3—Cs1B—O4^vii^	42.3 (2)	Al1^xx^—O4—Cs1B^ii^	76.75 (4)
O2^i^—Cs1B—O4^vii^	51.1 (3)	Cs1B^xxii^—O4—Cs1B^ii^	135.21 (5)
O2^iv^—Cs1B—O4^vii^	40.9 (2)	Cs1A^xix^—O4—Cs1B^ii^	136.11 (9)
O3^iv^—Cs1B—O4^vii^	87.7 (4)	Cs1B^xix^—O4—Cs1B^ii^	137.4 (3)
O3^i^—Cs1B—O4^vii^	73.4 (3)	Cs1B^xxiii^—O4—Cs1B^ii^	135.61 (3)
O2—Cs1B—O4^vii^	68.2 (2)	Cs1B—O4—Cs1B^ii^	1.30 (19)
O2^iii^—Cs1B—O4^vii^	104.2 (4)	Cs1B^i^—O4—Cs1B^ii^	2.1 (3)
O3^ii^—Cs1B—O4^vii^	108.65 (13)	Cs1A—O4—Cs1B^ii^	0.59 (8)

Symmetry codes: (i) −*y*, *x*−*y*, *z*; (ii) −*x*+*y*, −*x*, *z*; (iii) −*x*, −*x*+*y*, −*z*+3/2; (iv) *y*, *x*, −*z*+3/2; (v) *x*−*y*, −*y*, −*z*+3/2; (vi) −*y*, *x*−*y*−1, *z*; (vii) *y*, *x*−1, −*z*+3/2; (viii) *x*+1, *y*+1, *z*; (ix) −*y*, *x*−*y*+1, *z*; (x) *x*, *y*+1, *z*; (xi) −*x*+*y*+1, −*x*+1, *z*; (xii) *x*−*y*−1/3, *x*+1/3, −*z*+4/3; (xiii) *y*+2/3, −*x*+*y*+4/3, −*z*+4/3; (xiv) −*x*+2/3, −*y*+1/3, −*z*+4/3; (xv) *x*−1, *y*, *z*; (xvi) −*y*−1, *x*−*y*−1, *z*; (xvii) *x*−1, *y*−1, *z*; (xviii) −*x*+*y*−1, −*x*−1, *z*; (xix) *x*+1, *y*, *z*; (xx) *x*, *y*−1, *z*; (xxi) −*x*+*y*, −*x*−1, *z*; (xxii) −*x*+*y*+1, −*x*, *z*; (xxiii) −*y*+1, *x*−*y*, *z*.

### Caesium dialuminium arsenic(V) hexakis[hydrogen arsenate(V)] (CsAl2AsHAsO46) Hydrogen-bond geometry (Å, º)

**Table:**

*D*—H···*A*	*D*—H	H···*A*	*D*···*A*	*D*—H···*A*
O3—H···O4^vii^	0.88 (2)	1.94 (2)	2.7321 (18)	150 (3)

Symmetry code: (vii) *y*, *x*−1, −*z*+3/2.

### Caesium digallium arsenic(V) hexakis[hydrogen arsenate(V)] (CsGa2AsHAsO46) Crystal data

**Table:**

CsGa_2_As(HAsO_4_)_6_	*D*_x_ = 3.717 Mg m^−^^3^
*M**_r_* = 1186.84	Mo *K*α radiation, λ = 0.71073 Å
Trigonal, *R*3*c*:*H*	Cell parameters from 2582 reflections
*a* = 8.5199 (10) Å	θ = 2.9–32.6°
*c* = 50.608 (11) Å	µ = 15.18 mm^−^^1^
*V* = 3181.4 (10) Å^3^	*T* = 293 K
*Z* = 6	Pseudo-octahedra, colourless
*F*(000) = 3276	0.07 × 0.07 × 0.07 mm

### Caesium digallium arsenic(V) hexakis[hydrogen arsenate(V)] (CsGa2AsHAsO46) Data collection

**Table:**

Nonius KappaCCD single-crystal four-circle diffractometer	1134 reflections with *I* > 2σ(*I*)
Radiation source: fine-focus sealed tube	*R*_int_ = 0.018
φ and ω scans	θ_max_ = 32.6°, θ_min_ = 2.9°
Absorption correction: multi-scan (HKL SCALEPACK; Otwinowski *et al.*, 2003)	*h* = −12→12
*T*_min_ = 0.416, *T*_max_ = 0.416	*k* = −10→10
4712 measured reflections	*l* = −76→76
1293 independent reflections	

### Caesium digallium arsenic(V) hexakis[hydrogen arsenate(V)] (CsGa2AsHAsO46) Refinement

**Table:**

Refinement on *F*^2^	Secondary atom site location: difference Fourier map
Least-squares matrix: full	Hydrogen site location: difference Fourier map
*R*[*F*^2^ > 2σ(*F*^2^)] = 0.018	All H-atom parameters refined
*wR*(*F*^2^) = 0.041	*w* = 1/[σ^2^(*F*_o_^2^) + (0.0155*P*)^2^ + 13.7864*P*] where *P* = (*F*_o_^2^ + 2*F*_c_^2^)/3
*S* = 1.16	(Δ/σ)_max_ = 0.004
1293 reflections	Δρ_max_ = 0.86 e Å^−^^3^
65 parameters	Δρ_min_ = −0.72 e Å^−^^3^
2 restraints	Extinction correction: SHELXL-2016/6 (Sheldrick 2015), Fc^*^=kFc[1+0.001xFc^2^λ^3^/sin(2θ)]^-1/4^
Primary atom site location: structure-invariant direct methods	Extinction coefficient: 0.000080 (16)

### Caesium digallium arsenic(V) hexakis[hydrogen arsenate(V)] (CsGa2AsHAsO46) Special details

**Table:**

Geometry. All esds (except the esd in the dihedral angle between two l.s. planes) are estimated using the full covariance matrix. The cell esds are taken into account individually in the estimation of esds in distances, angles and torsion angles; correlations between esds in cell parameters are only used when they are defined by crystal symmetry. An approximate (isotropic) treatment of cell esds is used for estimating esds involving l.s. planes.

### Caesium digallium arsenic(V) hexakis[hydrogen arsenate(V)] (CsGa2AsHAsO46) Fractional atomic coordinates and isotropic or equivalent isotropic displacement parameters (Å^2^)

**Table:**

	*x*	*y*	*z*	*U*_iso_*/*U*_eq_	Occ. (<1)
Cs1A	0.000000	0.000000	0.750000	0.033 (3)	0.720 (3)
Cs1B	0.000000	−0.030 (5)	0.750000	0.0264 (19)	0.0932 (9)
Ga1	0.333333	0.666667	0.75610 (2)	0.00886 (8)	
As2	0.333333	0.666667	0.666667	0.00779 (10)	
As1	−0.44795 (3)	−0.41187 (3)	0.71128 (2)	0.00894 (6)	
O1	0.3969 (2)	−0.47002 (19)	0.68643 (3)	0.0119 (3)	
O2	−0.4545 (2)	−0.27504 (19)	0.73418 (3)	0.0113 (3)	
O3	−0.2351 (2)	−0.2915 (2)	0.69800 (4)	0.0203 (3)	
O4	0.4903 (2)	−0.1257 (2)	0.77880 (3)	0.0121 (3)	
H	−0.179 (5)	−0.350 (5)	0.7009 (7)	0.045 (11)*	

### Caesium digallium arsenic(V) hexakis[hydrogen arsenate(V)] (CsGa2AsHAsO46) Atomic displacement parameters (Å^2^)

**Table:**

	*U*^11^	*U*^22^	*U*^33^	*U*^12^	*U*^13^	*U*^23^
Cs1A	0.030 (4)	0.030 (4)	0.041 (2)	0.015 (2)	0.000	0.000
Cs1B	0.021 (6)	0.024 (4)	0.034 (5)	0.010 (3)	0.002 (2)	0.0011 (11)
Ga1	0.00933 (11)	0.00933 (11)	0.00791 (16)	0.00466 (6)	0.000	0.000
As2	0.00862 (13)	0.00862 (13)	0.00615 (19)	0.00431 (7)	0.000	0.000
As1	0.00989 (10)	0.00944 (10)	0.00872 (9)	0.00576 (8)	0.00007 (7)	0.00054 (7)
O1	0.0154 (7)	0.0121 (6)	0.0097 (6)	0.0081 (6)	−0.0034 (5)	0.0000 (5)
O2	0.0114 (6)	0.0108 (6)	0.0114 (6)	0.0054 (5)	0.0004 (5)	−0.0020 (5)
O3	0.0122 (7)	0.0210 (8)	0.0281 (9)	0.0086 (7)	0.0080 (6)	0.0067 (7)
O4	0.0143 (7)	0.0101 (6)	0.0143 (6)	0.0079 (6)	−0.0036 (5)	−0.0039 (5)

### Caesium digallium arsenic(V) hexakis[hydrogen arsenate(V)] (CsGa2AsHAsO46) Geometric parameters (Å, º)

**Table:**

Cs1A—Cs1B^i^	0.25 (5)	Cs1B—O2	3.451 (2)
Cs1A—Cs1B^ii^	0.25 (4)	Cs1B—O2^iii^	3.451 (2)
Cs1A—O2	3.4719 (16)	Cs1B—O3^ii^	3.65 (3)
Cs1A—O2^iii^	3.4719 (15)	Cs1B—O3^v^	3.65 (3)
Cs1A—O2^iv^	3.4719 (16)	Cs1B—O2^ii^	3.70 (4)
Cs1A—O2^ii^	3.4719 (15)	Cs1B—O2^v^	3.70 (4)
Cs1A—O2^v^	3.4719 (15)	Cs1B—O4^vi^	3.95 (4)
Cs1A—O2^i^	3.4719 (15)	Cs1B—O4^vii^	3.95 (4)
Cs1A—O3^iii^	3.4828 (19)	Cs1B—As1^iv^	4.043 (19)
Cs1A—O3^ii^	3.4828 (19)	Cs1B—As1^i^	4.043 (19)
Cs1A—O3^iv^	3.4829 (19)	Cs1B—As1^iii^	4.072 (14)
Cs1A—O3^v^	3.4829 (19)	Cs1B—As1	4.072 (14)
Cs1A—O3^i^	3.4829 (19)	Ga1—O2^viii^	1.9612 (14)
Cs1A—O3	3.483 (2)	Ga1—O2^ii^	1.9612 (15)
Cs1A—As1^iii^	4.1625 (5)	Ga1—O2^ix^	1.9612 (15)
Cs1A—As1^v^	4.1625 (5)	Ga1—O4^x^	1.9679 (15)
Cs1A—As1^iv^	4.1625 (5)	Ga1—O4^i^	1.9679 (15)
Cs1A—As1^ii^	4.1625 (5)	Ga1—O4^xi^	1.9679 (15)
Cs1A—As1^i^	4.1625 (5)	As2—O1^xii^	1.8109 (14)
Cs1A—As1	4.1626 (5)	As2—O1^xiii^	1.8109 (15)
Cs1B—Cs1B^i^	0.44 (8)	As2—O1^xiv^	1.8109 (14)
Cs1B—Cs1B^ii^	0.44 (7)	As2—O1^i^	1.8109 (15)
Cs1B—O2^iv^	3.28 (3)	As2—O1^xi^	1.8109 (14)
Cs1B—O2^i^	3.28 (3)	As2—O1^x^	1.8109 (14)
Cs1B—O3^iii^	3.383 (16)	As1—O2	1.6646 (14)
Cs1B—O3	3.383 (16)	As1—O4^iii^	1.6670 (15)
Cs1B—O3^iv^	3.436 (7)	As1—O1^xv^	1.7089 (14)
Cs1B—O3^i^	3.436 (7)	As1—O3	1.7125 (17)
			
Cs1B^i^—Cs1A—Cs1B^ii^	120.0 (3)	Cs1B^ii^—Cs1B—O4^vii^	153.66 (17)
Cs1B^i^—Cs1A—O2	153.53 (4)	O2^iv^—Cs1B—O4^vii^	52.0 (6)
Cs1B^ii^—Cs1A—O2	38.9 (2)	O2^i^—Cs1B—O4^vii^	43.4 (5)
Cs1B^i^—Cs1A—O2^iii^	38.93 (3)	O3^iii^—Cs1B—O4^vii^	42.8 (4)
Cs1B^ii^—Cs1A—O2^iii^	153.5 (2)	O3—Cs1B—O4^vii^	94.6 (9)
O2—Cs1A—O2^iii^	166.53 (5)	O3^iv^—Cs1B—O4^vii^	74.4 (6)
Cs1B^i^—Cs1A—O2^iv^	153.53 (4)	O3^i^—Cs1B—O4^vii^	90.2 (7)
Cs1B^ii^—Cs1A—O2^iv^	83.3 (3)	O2—Cs1B—O4^vii^	105.9 (9)
O2—Cs1A—O2^iv^	52.94 (5)	O2^iii^—Cs1B—O4^vii^	69.3 (5)
O2^iii^—Cs1A—O2^iv^	114.848 (17)	O3^ii^—Cs1B—O4^vii^	155.2 (6)
Cs1B^i^—Cs1A—O2^ii^	38.93 (3)	O3^v^—Cs1B—O4^vii^	108.8 (2)
Cs1B^ii^—Cs1A—O2^ii^	83.3 (3)	O2^ii^—Cs1B—O4^vii^	144.37 (3)
O2—Cs1A—O2^ii^	114.849 (17)	O2^v^—Cs1B—O4^vii^	145.20 (3)
O2^iii^—Cs1A—O2^ii^	77.86 (5)	O4^vi^—Cs1B—O4^vii^	51.9 (5)
O2^iv^—Cs1A—O2^ii^	166.53 (5)	Cs1B^i^—Cs1B—As1^iv^	139.7 (5)
Cs1B^i^—Cs1A—O2^v^	83.27 (2)	Cs1B^ii^—Cs1B—As1^iv^	90.7 (7)
Cs1B^ii^—Cs1A—O2^v^	38.9 (2)	O2^iv^—Cs1B—As1^iv^	23.43 (7)
O2—Cs1A—O2^v^	77.86 (5)	O2^i^—Cs1B—As1^iv^	104.5 (10)
O2^iii^—Cs1A—O2^v^	114.847 (17)	O3^iii^—Cs1B—As1^iv^	81.1 (5)
O2^iv^—Cs1A—O2^v^	114.848 (17)	O3—Cs1B—As1^iv^	80.2 (5)
O2^ii^—Cs1A—O2^v^	52.94 (5)	O3^iv^—Cs1B—As1^iv^	24.81 (16)
Cs1B^i^—Cs1A—O2^i^	83.27 (2)	O3^i^—Cs1B—As1^iv^	140.9 (11)
Cs1B^ii^—Cs1A—O2^i^	153.5 (2)	O2—Cs1B—As1^iv^	50.4 (2)
O2—Cs1A—O2^i^	114.848 (17)	O2^iii^—Cs1B—As1^iv^	126.7 (7)
O2^iii^—Cs1A—O2^i^	52.94 (5)	O3^ii^—Cs1B—As1^iv^	124.7 (3)
O2^iv^—Cs1A—O2^i^	77.86 (5)	O3^v^—Cs1B—As1^iv^	92.41 (13)
O2^ii^—Cs1A—O2^i^	114.848 (17)	O2^ii^—Cs1B—As1^iv^	141.3 (8)
O2^v^—Cs1A—O2^i^	166.53 (5)	O2^v^—Cs1B—As1^iv^	91.9 (3)
Cs1B^i^—Cs1A—O3^iii^	77.37 (3)	O4^vi^—Cs1B—As1^iv^	66.4 (5)
Cs1B^ii^—Cs1A—O3^iii^	130.09 (5)	O4^vii^—Cs1B—As1^iv^	66.1 (5)
O2—Cs1A—O3^iii^	127.26 (4)	Cs1B^i^—Cs1B—As1^i^	90.7 (5)
O2^iii^—Cs1A—O3^iii^	45.15 (4)	Cs1B^ii^—Cs1B—As1^i^	139.7 (4)
O2^iv^—Cs1A—O3^iii^	77.63 (4)	O2^iv^—Cs1B—As1^i^	104.5 (10)
O2^ii^—Cs1A—O3^iii^	111.42 (4)	O2^i^—Cs1B—As1^i^	23.43 (7)
O2^v^—Cs1A—O3^iii^	113.36 (4)	O3^iii^—Cs1B—As1^i^	80.2 (5)
O2^i^—Cs1A—O3^iii^	63.40 (4)	O3—Cs1B—As1^i^	81.1 (5)
Cs1B^i^—Cs1A—O3^ii^	77.37 (3)	O3^iv^—Cs1B—As1^i^	140.9 (11)
Cs1B^ii^—Cs1A—O3^ii^	64.82 (14)	O3^i^—Cs1B—As1^i^	24.81 (16)
O2—Cs1A—O3^ii^	77.64 (4)	O2—Cs1B—As1^i^	126.7 (7)
O2^iii^—Cs1A—O3^ii^	111.42 (4)	O2^iii^—Cs1B—As1^i^	50.4 (2)
O2^iv^—Cs1A—O3^ii^	127.26 (4)	O3^ii^—Cs1B—As1^i^	92.41 (13)
O2^ii^—Cs1A—O3^ii^	45.15 (4)	O3^v^—Cs1B—As1^i^	124.7 (3)
O2^v^—Cs1A—O3^ii^	63.40 (4)	O2^ii^—Cs1B—As1^i^	91.9 (3)
O2^i^—Cs1A—O3^ii^	113.36 (4)	O2^v^—Cs1B—As1^i^	141.3 (8)
O3^iii^—Cs1A—O3^ii^	154.75 (6)	O4^vi^—Cs1B—As1^i^	66.1 (5)
Cs1B^i^—Cs1A—O3^iv^	130.09 (3)	O4^vii^—Cs1B—As1^i^	66.4 (5)
Cs1B^ii^—Cs1A—O3^iv^	64.82 (15)	As1^iv^—Cs1B—As1^i^	126.9 (11)
O2—Cs1A—O3^iv^	63.40 (4)	Cs1B^i^—Cs1B—As1^iii^	83.1 (5)
O2^iii^—Cs1A—O3^iv^	113.36 (4)	Cs1B^ii^—Cs1B—As1^iii^	133.8 (4)
O2^iv^—Cs1A—O3^iv^	45.15 (4)	O2^iv^—Cs1B—As1^iii^	97.9 (8)
O2^ii^—Cs1A—O3^iv^	127.26 (4)	O2^i^—Cs1B—As1^iii^	50.8 (3)
O2^v^—Cs1A—O3^iv^	77.63 (4)	O3^iii^—Cs1B—As1^iii^	24.39 (9)
O2^i^—Cs1A—O3^iv^	111.42 (4)	O3—Cs1B—As1^iii^	132.1 (10)
O3^iii^—Cs1A—O3^iv^	69.12 (4)	O3^iv^—Cs1B—As1^iii^	95.2 (4)
O3^ii^—Cs1A—O3^iv^	129.64 (6)	O3^i^—Cs1B—As1^iii^	79.1 (3)
Cs1B^i^—Cs1A—O3^v^	64.82 (4)	O2—Cs1B—As1^iii^	152.4 (10)
Cs1B^ii^—Cs1A—O3^v^	77.37 (17)	O2^iii^—Cs1B—As1^iii^	23.78 (13)
O2—Cs1A—O3^v^	111.41 (4)	O3^ii^—Cs1B—As1^iii^	131.7 (5)
O2^iii^—Cs1A—O3^v^	77.64 (4)	O3^v^—Cs1B—As1^iii^	77.69 (14)
O2^iv^—Cs1A—O3^v^	113.36 (4)	O2^ii^—Cs1B—As1^iii^	96.6 (4)
O2^ii^—Cs1A—O3^v^	63.40 (4)	O2^v^—Cs1B—As1^iii^	119.1 (6)
O2^v^—Cs1A—O3^v^	45.15 (4)	O4^vi^—Cs1B—As1^iii^	94.1 (8)
O2^i^—Cs1A—O3^v^	127.26 (4)	O4^vii^—Cs1B—As1^iii^	48.3 (3)
O3^iii^—Cs1A—O3^v^	69.12 (4)	As1^iv^—Cs1B—As1^iii^	103.2 (6)
O3^ii^—Cs1A—O3^v^	99.81 (6)	As1^i^—Cs1B—As1^iii^	58.3 (3)
O3^iv^—Cs1A—O3^v^	69.12 (4)	Cs1B^i^—Cs1B—As1	133.8 (6)
Cs1B^i^—Cs1A—O3^i^	64.82 (3)	Cs1B^ii^—Cs1B—As1	83.1 (7)
Cs1B^ii^—Cs1A—O3^i^	130.09 (5)	O2^iv^—Cs1B—As1	50.8 (3)
O2—Cs1A—O3^i^	113.36 (4)	O2^i^—Cs1B—As1	97.9 (8)
O2^iii^—Cs1A—O3^i^	63.40 (4)	O3^iii^—Cs1B—As1	132.1 (10)
O2^iv^—Cs1A—O3^i^	111.42 (4)	O3—Cs1B—As1	24.39 (9)
O2^ii^—Cs1A—O3^i^	77.64 (4)	O3^iv^—Cs1B—As1	79.2 (3)
O2^v^—Cs1A—O3^i^	127.26 (4)	O3^i^—Cs1B—As1	95.2 (4)
O2^i^—Cs1A—O3^i^	45.15 (4)	O2—Cs1B—As1	23.78 (13)
O3^iii^—Cs1A—O3^i^	99.81 (6)	O2^iii^—Cs1B—As1	152.4 (10)
O3^ii^—Cs1A—O3^i^	69.12 (5)	O3^ii^—Cs1B—As1	77.69 (14)
O3^iv^—Cs1A—O3^i^	154.75 (5)	O3^v^—Cs1B—As1	131.7 (5)
O3^v^—Cs1A—O3^i^	129.64 (6)	O2^ii^—Cs1B—As1	119.1 (6)
Cs1B^i^—Cs1A—O3	130.09 (3)	O2^v^—Cs1B—As1	96.6 (4)
Cs1B^ii^—Cs1A—O3	77.37 (17)	O4^vi^—Cs1B—As1	48.3 (3)
O2—Cs1A—O3	45.15 (4)	O4^vii^—Cs1B—As1	94.1 (8)
O2^iii^—Cs1A—O3	127.26 (4)	As1^iv^—Cs1B—As1	58.3 (3)
O2^iv^—Cs1A—O3	63.40 (4)	As1^i^—Cs1B—As1	103.2 (6)
O2^ii^—Cs1A—O3	113.36 (4)	As1^iii^—Cs1B—As1	141.4 (11)
O2^v^—Cs1A—O3	111.42 (4)	O2^viii^—Ga1—O2^ii^	91.15 (6)
O2^i^—Cs1A—O3	77.63 (4)	O2^viii^—Ga1—O2^ix^	91.15 (6)
O3^iii^—Cs1A—O3	129.64 (6)	O2^ii^—Ga1—O2^ix^	91.15 (6)
O3^ii^—Cs1A—O3	69.12 (5)	O2^viii^—Ga1—O4^x^	90.84 (6)
O3^iv^—Cs1A—O3	99.81 (6)	O2^ii^—Ga1—O4^x^	178.00 (6)
O3^v^—Cs1A—O3	154.75 (6)	O2^ix^—Ga1—O4^x^	88.66 (6)
O3^i^—Cs1A—O3	69.12 (5)	O2^viii^—Ga1—O4^i^	88.66 (6)
Cs1B^i^—Cs1A—As1^iii^	60.306 (12)	O2^ii^—Ga1—O4^i^	90.84 (6)
Cs1B^ii^—Cs1A—As1^iii^	151.64 (4)	O2^ix^—Ga1—O4^i^	178.00 (6)
O2—Cs1A—As1^iii^	146.15 (2)	O4^x^—Ga1—O4^i^	89.35 (6)
O2^iii^—Cs1A—As1^iii^	22.98 (2)	O2^viii^—Ga1—O4^xi^	178.00 (6)
O2^iv^—Cs1A—As1^iii^	93.23 (2)	O2^ii^—Ga1—O4^xi^	88.66 (6)
O2^ii^—Cs1A—As1^iii^	98.62 (2)	O2^ix^—Ga1—O4^xi^	90.84 (6)
O2^v^—Cs1A—As1^iii^	122.59 (2)	O4^x^—Ga1—O4^xi^	89.35 (6)
O2^i^—Cs1A—As1^iii^	49.10 (2)	O4^i^—Ga1—O4^xi^	89.35 (7)
O3^iii^—Cs1A—As1^iii^	23.83 (3)	O2^viii^—Ga1—Cs1B^x^	34.15 (10)
O3^ii^—Cs1A—As1^iii^	134.28 (3)	O2^ii^—Ga1—Cs1B^x^	99.2 (3)
O3^iv^—Cs1A—As1^iii^	92.90 (3)	O2^ix^—Ga1—Cs1B^x^	123.8 (2)
O3^v^—Cs1A—As1^iii^	78.30 (3)	O4^x^—Ga1—Cs1B^x^	82.5 (2)
O3^i^—Cs1A—As1^iii^	77.37 (3)	O4^i^—Ga1—Cs1B^x^	55.68 (16)
O3—Cs1A—As1^iii^	125.98 (3)	O4^xi^—Ga1—Cs1B^x^	143.97 (12)
Cs1B^i^—Cs1A—As1^v^	67.383 (11)	O2^viii^—Ga1—Cs1B^i^	123.79 (17)
Cs1B^ii^—Cs1A—As1^v^	60.3 (2)	O2^ii^—Ga1—Cs1B^i^	34.15 (9)
O2—Cs1A—As1^v^	98.62 (2)	O2^ix^—Ga1—Cs1B^i^	99.2 (2)
O2^iii^—Cs1A—As1^v^	93.23 (2)	O4^x^—Ga1—Cs1B^i^	143.97 (10)
O2^iv^—Cs1A—As1^v^	122.59 (2)	O4^i^—Ga1—Cs1B^i^	82.5 (2)
O2^ii^—Cs1A—As1^v^	49.10 (2)	O4^xi^—Ga1—Cs1B^i^	55.68 (16)
O2^v^—Cs1A—As1^v^	22.98 (2)	Cs1B^x^—Ga1—Cs1B^i^	119.57 (2)
O2^i^—Cs1A—As1^v^	146.15 (2)	O2^viii^—Ga1—Cs1B^xi^	99.2 (3)
O3^iii^—Cs1A—As1^v^	92.90 (3)	O2^ii^—Ga1—Cs1B^xi^	123.79 (19)
O3^ii^—Cs1A—As1^v^	77.37 (3)	O2^ix^—Ga1—Cs1B^xi^	34.15 (12)
O3^iv^—Cs1A—As1^v^	78.29 (3)	O4^x^—Ga1—Cs1B^xi^	55.68 (19)
O3^v^—Cs1A—As1^v^	23.82 (3)	O4^i^—Ga1—Cs1B^xi^	143.97 (13)
O3^i^—Cs1A—As1^v^	125.98 (3)	O4^xi^—Ga1—Cs1B^xi^	82.5 (3)
O3—Cs1A—As1^v^	134.28 (3)	Cs1B^x^—Ga1—Cs1B^xi^	119.575 (14)
As1^iii^—Cs1A—As1^v^	99.647 (7)	Cs1B^i^—Ga1—Cs1B^xi^	119.575 (5)
Cs1B^i^—Cs1A—As1^iv^	151.635 (12)	O2^viii^—Ga1—Cs1A^x^	33.70 (4)
Cs1B^ii^—Cs1A—As1^iv^	67.4 (2)	O2^ii^—Ga1—Cs1A^x^	100.57 (4)
O2—Cs1A—As1^iv^	49.10 (2)	O2^ix^—Ga1—Cs1A^x^	122.84 (4)
O2^iii^—Cs1A—As1^iv^	122.59 (2)	O4^x^—Ga1—Cs1A^x^	81.19 (4)
O2^iv^—Cs1A—As1^iv^	22.98 (2)	O4^i^—Ga1—Cs1A^x^	56.58 (4)
O2^ii^—Cs1A—As1^iv^	146.15 (2)	O4^xi^—Ga1—Cs1A^x^	144.45 (5)
O2^v^—Cs1A—As1^iv^	93.23 (2)	Cs1B^x^—Ga1—Cs1A^x^	1.6 (3)
O2^i^—Cs1A—As1^iv^	98.62 (2)	Cs1B^i^—Ga1—Cs1A^x^	121.1 (3)
O3^iii^—Cs1A—As1^iv^	78.30 (3)	Cs1B^xi^—Ga1—Cs1A^x^	118.1 (3)
O3^ii^—Cs1A—As1^iv^	125.98 (3)	O2^viii^—Ga1—Cs1A	122.84 (4)
O3^iv^—Cs1A—As1^iv^	23.83 (3)	O2^ii^—Ga1—Cs1A	33.70 (4)
O3^v^—Cs1A—As1^iv^	92.90 (3)	O2^ix^—Ga1—Cs1A	100.57 (4)
O3^i^—Cs1A—As1^iv^	134.28 (3)	O4^x^—Ga1—Cs1A	144.45 (5)
O3—Cs1A—As1^iv^	77.37 (3)	O4^i^—Ga1—Cs1A	81.19 (4)
As1^iii^—Cs1A—As1^iv^	99.647 (7)	O4^xi^—Ga1—Cs1A	56.58 (4)
As1^v^—Cs1A—As1^iv^	99.647 (8)	Cs1B^x^—Ga1—Cs1A	118.1 (3)
Cs1B^i^—Cs1A—As1^ii^	60.306 (11)	Cs1B^i^—Ga1—Cs1A	1.6 (3)
Cs1B^ii^—Cs1A—As1^ii^	67.4 (2)	Cs1B^xi^—Ga1—Cs1A	121.1 (3)
O2—Cs1A—As1^ii^	93.23 (2)	Cs1A^x^—Ga1—Cs1A	119.612 (1)
O2^iii^—Cs1A—As1^ii^	98.62 (2)	O2^viii^—Ga1—Cs1A^ix^	100.57 (4)
O2^iv^—Cs1A—As1^ii^	146.15 (2)	O2^ii^—Ga1—Cs1A^ix^	122.84 (4)
O2^ii^—Cs1A—As1^ii^	22.98 (2)	O2^ix^—Ga1—Cs1A^ix^	33.70 (4)
O2^v^—Cs1A—As1^ii^	49.10 (2)	O4^x^—Ga1—Cs1A^ix^	56.58 (4)
O2^i^—Cs1A—As1^ii^	122.59 (2)	O4^i^—Ga1—Cs1A^ix^	144.45 (5)
O3^iii^—Cs1A—As1^ii^	134.28 (3)	O4^xi^—Ga1—Cs1A^ix^	81.19 (4)
O3^ii^—Cs1A—As1^ii^	23.83 (3)	Cs1B^x^—Ga1—Cs1A^ix^	121.1 (3)
O3^iv^—Cs1A—As1^ii^	125.98 (3)	Cs1B^i^—Ga1—Cs1A^ix^	118.1 (3)
O3^v^—Cs1A—As1^ii^	77.37 (3)	Cs1B^xi^—Ga1—Cs1A^ix^	1.6 (3)
O3^i^—Cs1A—As1^ii^	78.30 (3)	Cs1A^x^—Ga1—Cs1A^ix^	119.612 (1)
O3—Cs1A—As1^ii^	92.90 (3)	Cs1A—Ga1—Cs1A^ix^	119.612 (1)
As1^iii^—Cs1A—As1^ii^	120.612 (7)	O1^xii^—As2—O1^xiii^	92.46 (6)
As1^v^—Cs1A—As1^ii^	56.729 (13)	O1^xii^—As2—O1^xiv^	92.45 (6)
As1^iv^—Cs1A—As1^ii^	134.766 (6)	O1^xiii^—As2—O1^xiv^	92.45 (6)
Cs1B^i^—Cs1A—As1^i^	67.383 (6)	O1^xii^—As2—O1^i^	87.55 (6)
Cs1B^ii^—Cs1A—As1^i^	151.64 (4)	O1^xiii^—As2—O1^i^	180.00 (12)
O2—Cs1A—As1^i^	122.59 (2)	O1^xiv^—As2—O1^i^	87.55 (6)
O2^iii^—Cs1A—As1^i^	49.10 (2)	O1^xii^—As2—O1^xi^	180.0
O2^iv^—Cs1A—As1^i^	98.62 (2)	O1^xiii^—As2—O1^xi^	87.55 (6)
O2^ii^—Cs1A—As1^i^	93.23 (2)	O1^xiv^—As2—O1^xi^	87.55 (6)
O2^v^—Cs1A—As1^i^	146.15 (2)	O1^i^—As2—O1^xi^	92.45 (6)
O2^i^—Cs1A—As1^i^	22.98 (2)	O1^xii^—As2—O1^x^	87.55 (6)
O3^iii^—Cs1A—As1^i^	77.37 (3)	O1^xiii^—As2—O1^x^	87.55 (6)
O3^ii^—Cs1A—As1^i^	92.90 (3)	O1^xiv^—As2—O1^x^	180.00 (8)
O3^iv^—Cs1A—As1^i^	134.29 (3)	O1^i^—As2—O1^x^	92.45 (6)
O3^v^—Cs1A—As1^i^	125.98 (3)	O1^xi^—As2—O1^x^	92.45 (6)
O3^i^—Cs1A—As1^i^	23.82 (3)	O2—As1—O4^iii^	117.09 (7)
O3—Cs1A—As1^i^	78.30 (3)	O2—As1—O1^xv^	115.06 (7)
As1^iii^—Cs1A—As1^i^	56.729 (13)	O4^iii^—As1—O1^xv^	100.76 (7)
As1^v^—Cs1A—As1^i^	134.766 (7)	O2—As1—O3	104.49 (8)
As1^iv^—Cs1A—As1^i^	120.612 (7)	O4^iii^—As1—O3	110.80 (8)
As1^ii^—Cs1A—As1^i^	99.647 (9)	O1^xv^—As1—O3	108.60 (8)
Cs1B^i^—Cs1A—As1	151.634 (13)	O2—As1—Cs1B^ii^	51.6 (5)
Cs1B^ii^—Cs1A—As1	60.3 (2)	O4^iii^—As1—Cs1B^ii^	113.4 (3)
O2—Cs1A—As1	22.98 (2)	O1^xv^—As1—Cs1B^ii^	145.8 (3)
O2^iii^—Cs1A—As1	146.15 (2)	O3—As1—Cs1B^ii^	57.4 (4)
O2^iv^—Cs1A—As1	49.10 (2)	O2—As1—Cs1B	56.7 (4)
O2^ii^—Cs1A—As1	122.58 (2)	O4^iii^—As1—Cs1B	108.4 (6)
O2^v^—Cs1A—As1	98.62 (2)	O1^xv^—As1—Cs1B	150.0 (5)
O2^i^—Cs1A—As1	93.23 (2)	O3—As1—Cs1B	54.65 (13)
O3^iii^—Cs1A—As1	125.98 (3)	Cs1B^ii^—As1—Cs1B	6.2 (11)
O3^ii^—Cs1A—As1	78.29 (3)	O2—As1—Cs1A	54.52 (5)
O3^iv^—Cs1A—As1	77.37 (3)	O4^iii^—As1—Cs1A	111.50 (5)
O3^v^—Cs1A—As1	134.29 (3)	O1^xv^—As1—Cs1A	147.27 (5)
O3^i^—Cs1A—As1	92.89 (3)	O3—As1—Cs1A	55.24 (6)
O3—Cs1A—As1	23.82 (3)	Cs1B^ii^—As1—Cs1A	3.1 (5)
As1^iii^—Cs1A—As1	134.766 (6)	Cs1B—As1—Cs1A	3.3 (6)
As1^v^—Cs1A—As1	120.613 (7)	O2—As1—Cs1B^i^	55.25 (13)
As1^iv^—Cs1A—As1	56.731 (13)	O4^iii^—As1—Cs1B^i^	112.60 (18)
As1^ii^—Cs1A—As1	99.647 (9)	O1^xv^—As1—Cs1B^i^	146.0 (2)
As1^i^—Cs1A—As1	99.646 (9)	O3—As1—Cs1B^i^	53.9 (2)
Cs1B^i^—Cs1B—Cs1B^ii^	60.00 (6)	Cs1B^ii^—As1—Cs1B^i^	3.7 (6)
Cs1B^i^—Cs1B—O2^iv^	162.9 (4)	Cs1B—As1—Cs1B^i^	4.2 (7)
Cs1B^ii^—Cs1B—O2^iv^	109.7 (7)	Cs1A—As1—Cs1B^i^	1.6 (3)
Cs1B^i^—Cs1B—O2^i^	109.7 (5)	O2—As1—Cs1B^xvi^	93.88 (14)
Cs1B^ii^—Cs1B—O2^i^	162.9 (3)	O4^iii^—As1—Cs1B^xvi^	42.74 (6)
O2^iv^—Cs1B—O2^i^	83.4 (10)	O1^xv^—As1—Cs1B^xvi^	79.72 (19)
Cs1B^i^—Cs1B—O3^iii^	93.3 (6)	O3—As1—Cs1B^xvi^	153.38 (6)
Cs1B^ii^—Cs1B—O3^iii^	124.8 (5)	Cs1B^ii^—As1—Cs1B^xvi^	127.8 (3)
O2^iv^—Cs1B—O3^iii^	81.8 (7)	Cs1B—As1—Cs1B^xvi^	127.1 (4)
O2^i^—Cs1B—O3^iii^	66.5 (5)	Cs1A—As1—Cs1B^xvi^	128.4 (2)
Cs1B^i^—Cs1B—O3	124.8 (6)	Cs1B^i^—As1—Cs1B^xvi^	129.96 (7)
Cs1B^ii^—Cs1B—O3	93.3 (7)	O2—As1—Cs1A^xvii^	94.63 (5)
O2^iv^—Cs1B—O3	66.5 (5)	O4^iii^—As1—Cs1A^xvii^	42.93 (5)
O2^i^—Cs1B—O3	81.8 (7)	O1^xv^—As1—Cs1A^xvii^	78.68 (5)
O3^iii^—Cs1B—O3	137.4 (14)	O3—As1—Cs1A^xvii^	153.41 (6)
Cs1B^i^—Cs1B—O3^iv^	116.2 (7)	Cs1B^ii^—As1—Cs1A^xvii^	128.92 (12)
Cs1B^ii^—Cs1B—O3^iv^	79.3 (8)	Cs1B—As1—Cs1A^xvii^	128.2 (2)
O2^iv^—Cs1B—O3^iv^	46.8 (3)	Cs1A—As1—Cs1A^xvii^	129.479 (13)
O2^i^—Cs1B—O3^iv^	117.7 (11)	Cs1B^i^—As1—Cs1A^xvii^	131.1 (3)
O3^iii^—Cs1B—O3^iv^	70.8 (3)	Cs1B^xvi^—As1—Cs1A^xvii^	1.1 (2)
O3—Cs1B—O3^iv^	102.8 (5)	O2—As1—Cs1B^xviii^	93.5 (2)
Cs1B^i^—Cs1B—O3^i^	79.3 (6)	O4^iii^—As1—Cs1B^xviii^	45.4 (4)
Cs1B^ii^—Cs1B—O3^i^	116.2 (5)	O1^xv^—As1—Cs1B^xviii^	77.2 (3)
O2^iv^—Cs1B—O3^i^	117.7 (11)	O3—As1—Cs1B^xviii^	155.7 (4)
O2^i^—Cs1B—O3^i^	46.8 (3)	Cs1B^ii^—As1—Cs1B^xviii^	129.53 (5)
O3^iii^—Cs1B—O3^i^	102.8 (5)	Cs1B—As1—Cs1B^xviii^	129.12 (6)
O3—Cs1B—O3^i^	70.8 (3)	Cs1A—As1—Cs1B^xviii^	130.25 (12)
O3^iv^—Cs1B—O3^i^	163.0 (14)	Cs1B^i^—As1—Cs1B^xviii^	131.8 (4)
Cs1B^i^—Cs1B—O2	121.6 (7)	Cs1B^xvi^—As1—Cs1B^xviii^	3.0 (5)
Cs1B^ii^—Cs1B—O2	63.4 (9)	Cs1A^xvii^—As1—Cs1B^xviii^	2.5 (4)
O2^iv^—Cs1B—O2	54.7 (3)	O2—As1—Cs1B^xvii^	96.5 (3)
O2^i^—Cs1B—O2	120.8 (10)	O4^iii^—As1—Cs1B^xvii^	40.7 (4)
O3^iii^—Cs1B—O2	131.5 (6)	O1^xv^—As1—Cs1B^xvii^	79.23 (14)
O3—Cs1B—O2	45.98 (12)	O3—As1—Cs1B^xvii^	151.1 (4)
O3^iv^—Cs1B—O2	64.09 (9)	Cs1B^ii^—As1—Cs1B^xvii^	129.27 (4)
O3^i^—Cs1B—O2	115.1 (2)	Cs1B—As1—Cs1B^xvii^	128.2 (3)
Cs1B^i^—Cs1B—O2^iii^	63.4 (7)	Cs1A—As1—Cs1B^xvii^	129.66 (3)
Cs1B^ii^—Cs1B—O2^iii^	121.6 (5)	Cs1B^i^—As1—Cs1B^xvii^	131.2 (3)
O2^iv^—Cs1B—O2^iii^	120.8 (10)	Cs1B^xvi^—As1—Cs1B^xvii^	2.7 (4)
O2^i^—Cs1B—O2^iii^	54.7 (3)	Cs1A^xvii^—As1—Cs1B^xvii^	2.3 (4)
O3^iii^—Cs1B—O2^iii^	45.98 (12)	Cs1B^xviii^—As1—Cs1B^xvii^	4.7 (8)
O3—Cs1B—O2^iii^	131.5 (6)	As1^xix^—O1—As2^xx^	131.05 (8)
O3^iv^—Cs1B—O2^iii^	115.1 (2)	As1^xix^—O1—Cs1B^vii^	80.6 (3)
O3^i^—Cs1B—O2^iii^	64.09 (9)	As2^xx^—O1—Cs1B^vii^	130.0 (2)
O2—Cs1B—O2^iii^	174.9 (14)	As1—O2—Ga1^xvii^	124.47 (8)
Cs1B^i^—Cs1B—O3^ii^	57.6 (4)	As1—O2—Cs1B^ii^	105.0 (5)
Cs1B^ii^—Cs1B—O3^ii^	49.5 (3)	Ga1^xvii^—O2—Cs1B^ii^	126.2 (4)
O2^iv^—Cs1B—O3^ii^	128.02 (8)	As1—O2—Cs1B	99.5 (5)
O2^i^—Cs1B—O3^ii^	113.88 (5)	Ga1^xvii^—O2—Cs1B	129.4 (2)
O3^iii^—Cs1B—O3^ii^	150.1 (9)	Cs1B^ii^—O2—Cs1B	6.9 (12)
O3—Cs1B—O3^ii^	68.2 (2)	As1—O2—Cs1A	102.50 (6)
O3^iv^—Cs1B—O3^ii^	125.6 (7)	Ga1^xvii^—O2—Cs1A	128.04 (6)
O3^i^—Cs1B—O3^ii^	67.7 (3)	Cs1B^ii^—O2—Cs1A	2.8 (5)
O2—Cs1B—O3^ii^	75.7 (4)	Cs1B—O2—Cs1A	4.2 (7)
O2^iii^—Cs1B—O3^ii^	108.0 (6)	As1—O2—Cs1B^i^	103.06 (11)
Cs1B^i^—Cs1B—O3^v^	49.5 (4)	Ga1^xvii^—O2—Cs1B^i^	128.20 (6)
Cs1B^ii^—Cs1B—O3^v^	57.6 (3)	Cs1B^ii^—O2—Cs1B^i^	2.0 (4)
O2^iv^—Cs1B—O3^v^	113.88 (5)	Cs1B—O2—Cs1B^i^	5.8 (10)
O2^i^—Cs1B—O3^v^	128.02 (8)	Cs1A—O2—Cs1B^i^	1.8 (3)
O3^iii^—Cs1B—O3^v^	68.2 (2)	As1—O3—Cs1B	100.96 (8)
O3—Cs1B—O3^v^	150.1 (8)	As1—O3—Cs1B^ii^	97.8 (5)
O3^iv^—Cs1B—O3^v^	67.7 (3)	Cs1B—O3—Cs1B^ii^	7.3 (13)
O3^i^—Cs1B—O3^v^	125.6 (7)	As1—O3—Cs1A	100.94 (7)
O2—Cs1B—O3^v^	107.9 (6)	Cs1B—O3—Cs1A	3.9 (7)
O2^iii^—Cs1B—O3^v^	75.7 (4)	Cs1B^ii^—O3—Cs1A	4.1 (7)
O3^ii^—Cs1B—O3^v^	93.7 (10)	As1—O3—Cs1B^i^	103.8 (5)
Cs1B^i^—Cs1B—O2^ii^	15.07 (4)	Cs1B—O3—Cs1B^i^	5.7 (10)
Cs1B^ii^—Cs1B—O2^ii^	52.57 (10)	Cs1B^ii^—O3—Cs1B^i^	6.2 (10)
O2^iv^—Cs1B—O2^ii^	162.2 (7)	Cs1A—O3—Cs1B^i^	3.1 (5)
O2^i^—Cs1B—O2^ii^	113.8 (2)	As1^iii^—O4—Ga1^xx^	126.05 (8)
O3^iii^—Cs1B—O2^ii^	108.3 (6)	As1^iii^—O4—Cs1B^xxi^	120.63 (19)
O3—Cs1B—O2^ii^	110.2 (6)	Ga1^xx^—O4—Cs1B^xxi^	100.03 (7)
O3^iv^—Cs1B—O2^ii^	121.6 (10)	As1^iii^—O4—Cs1A^xix^	121.31 (6)
O3^i^—Cs1B—O2^ii^	75.2 (4)	Ga1^xx^—O4—Cs1A^xix^	100.29 (5)
O2—Cs1B—O2^ii^	109.7 (9)	Cs1B^xxi^—O4—Cs1A^xix^	1.5 (3)
O2^iii^—Cs1B—O2^ii^	75.1 (5)	As1^iii^—O4—Cs1B^xix^	124.5 (6)
O3^ii^—Cs1B—O2^ii^	42.6 (4)	Ga1^xx^—O4—Cs1B^xix^	98.2 (4)
O3^v^—Cs1B—O2^ii^	59.6 (6)	Cs1B^xxi^—O4—Cs1B^xix^	4.5 (8)
Cs1B^i^—Cs1B—O2^v^	52.6 (3)	Cs1A^xix^—O4—Cs1B^xix^	3.3 (5)
Cs1B^ii^—Cs1B—O2^v^	15.07 (11)	As1^iii^—O4—Cs1B^xxii^	118.8 (4)
O2^iv^—Cs1B—O2^v^	113.8 (2)	Ga1^xx^—O4—Cs1B^xxii^	102.6 (4)
O2^i^—Cs1B—O2^v^	162.2 (7)	Cs1B^xxi^—O4—Cs1B^xxii^	2.6 (4)
O3^iii^—Cs1B—O2^v^	110.2 (6)	Cs1A^xix^—O4—Cs1B^xxii^	2.6 (4)
O3—Cs1B—O2^v^	108.3 (6)	Cs1B^xix^—O4—Cs1B^xxii^	5.7 (10)
O3^iv^—Cs1B—O2^v^	75.2 (4)	As1^iii^—O4—Cs1B	52.6 (4)
O3^i^—Cs1B—O2^v^	121.6 (10)	Ga1^xx^—O4—Cs1B	73.8 (4)
O2—Cs1B—O2^v^	75.1 (5)	Cs1B^xxi^—O4—Cs1B	134.69 (14)
O2^iii^—Cs1B—O2^v^	109.7 (9)	Cs1A^xix^—O4—Cs1B	136.21 (14)
O3^ii^—Cs1B—O2^v^	59.6 (6)	Cs1B^xix^—O4—Cs1B	138.2 (4)
O3^v^—Cs1B—O2^v^	42.6 (4)	Cs1B^xxii^—O4—Cs1B	135.56 (6)
O2^ii^—Cs1B—O2^v^	49.4 (6)	As1^iii^—O4—Cs1B^i^	48.6 (3)
Cs1B^i^—Cs1B—O4^vi^	153.66 (8)	Ga1^xx^—O4—Cs1B^i^	78.2 (4)
Cs1B^ii^—Cs1B—O4^vi^	131.4 (4)	Cs1B^xxi^—O4—Cs1B^i^	132.1 (6)
O2^iv^—Cs1B—O4^vi^	43.4 (5)	Cs1A^xix^—O4—Cs1B^i^	133.6 (4)
O2^i^—Cs1B—O4^vi^	52.0 (6)	Cs1B^xix^—O4—Cs1B^i^	135.86 (5)
O3^iii^—Cs1B—O4^vi^	94.6 (9)	Cs1B^xxii^—O4—Cs1B^i^	132.7 (5)
O3—Cs1B—O4^vi^	42.8 (4)	Cs1B—O4—Cs1B^i^	5.1 (9)
O3^iv^—Cs1B—O4^vi^	90.2 (7)	As1^iii^—O4—Cs1A	50.49 (4)
O3^i^—Cs1B—O4^vi^	74.4 (6)	Ga1^xx^—O4—Cs1A	76.01 (4)
O2—Cs1B—O4^vi^	69.3 (5)	Cs1B^xxi^—O4—Cs1A	133.9 (3)
O2^iii^—Cs1B—O4^vi^	105.9 (9)	Cs1A^xix^—O4—Cs1A	135.46 (4)
O3^ii^—Cs1B—O4^vi^	108.8 (2)	Cs1B^xix^—O4—Cs1A	137.6 (3)
O3^v^—Cs1B—O4^vi^	155.2 (6)	Cs1B^xxii^—O4—Cs1A	134.68 (14)
O2^ii^—Cs1B—O4^vi^	145.19 (3)	Cs1B—O4—Cs1A	2.3 (4)
O2^v^—Cs1B—O4^vi^	144.37 (3)	Cs1B^i^—O4—Cs1A	2.8 (5)
Cs1B^i^—Cs1B—O4^vii^	131.4 (2)		

Symmetry codes: (i) −*y*, *x*−*y*, *z*; (ii) −*x*+*y*, −*x*, *z*; (iii) −*x*, −*x*+*y*, −*z*+3/2; (iv) *y*, *x*, −*z*+3/2; (v) *x*−*y*, −*y*, −*z*+3/2; (vi) *y*, *x*−1, −*z*+3/2; (vii) −*y*, *x*−*y*−1, *z*; (viii) −*y*, *x*−*y*+1, *z*; (ix) *x*+1, *y*+1, *z*; (x) *x*, *y*+1, *z*; (xi) −*x*+*y*+1, −*x*+1, *z*; (xii) *x*−*y*−1/3, *x*+1/3, −*z*+4/3; (xiii) *y*+2/3, −*x*+*y*+4/3, −*z*+4/3; (xiv) −*x*+2/3, −*y*+1/3, −*z*+4/3; (xv) *x*−1, *y*, *z*; (xvi) −*y*−1, *x*−*y*−1, *z*; (xvii) *x*−1, *y*−1, *z*; (xviii) −*x*+*y*−1, −*x*−1, *z*; (xix) *x*+1, *y*, *z*; (xx) *x*, *y*−1, *z*; (xxi) −*x*+*y*+1, −*x*, *z*; (xxii) −*y*+1, *x*−*y*, *z*.

### Caesium digallium arsenic(V) hexakis[hydrogen arsenate(V)] (CsGa2AsHAsO46) Hydrogen-bond geometry (Å, º)

**Table:**

*D*—H···*A*	*D*—H	H···*A*	*D*···*A*	*D*—H···*A*
O3—H···O4^vi^	0.86 (2)	1.93 (2)	2.727 (2)	154 (3)

Symmetry code: (vi) *y*, *x*−1, −*z*+3/2.

### Thallium digallium arsenic(V) hexakis[hydrogen arsenate(V)] (TlGa2AsHAsO46) Crystal data

**Table:**

TlGa_2_As(HAsO_4_)_6_	*D*_x_ = 3.965 Mg m^−^^3^
*M**_r_* = 1258.30	Mo *K*α radiation, λ = 0.71073 Å
Trigonal, *R*3*c*:*H*	Cell parameters from 2570 reflections
*a* = 8.484 (1) Å	θ = 2.4–32.6°
*c* = 50.724 (11) Å	µ = 21.18 mm^−^^1^
*V* = 3161.9 (10) Å^3^	*T* = 293 K
*Z* = 6	Large pseudo-octahedra, colourless
*F*(000) = 3432	0.08 × 0.07 × 0.05 mm

### Thallium digallium arsenic(V) hexakis[hydrogen arsenate(V)] (TlGa2AsHAsO46) Data collection

**Table:**

Nonius KappaCCD single-crystal four-circle diffractometer	1129 reflections with *I* > 2σ(*I*)
Radiation source: fine-focus sealed tube	*R*_int_ = 0.024
φ and ω scans	θ_max_ = 32.6°, θ_min_ = 2.4°
Absorption correction: multi-scan HKL SCALEPACK (Otwinowski *et al.*, 2003)	*h* = −12→12
*T*_min_ = 0.200, *T*_max_ = 0.347	*k* = −10→10
4682 measured reflections	*l* = −76→76
1285 independent reflections	

### Thallium digallium arsenic(V) hexakis[hydrogen arsenate(V)] (TlGa2AsHAsO46) Refinement

**Table:**

Refinement on *F*^2^	Secondary atom site location: difference Fourier map
Least-squares matrix: full	Hydrogen site location: difference Fourier map
*R*[*F*^2^ > 2σ(*F*^2^)] = 0.018	All H-atom parameters refined
*wR*(*F*^2^) = 0.041	*w* = 1/[σ^2^(*F*_o_^2^) + (0.0174*P*)^2^ + 10.0458*P*] where *P* = (*F*_o_^2^ + 2*F*_c_^2^)/3
*S* = 1.10	(Δ/σ)_max_ = 0.005
1285 reflections	Δρ_max_ = 0.74 e Å^−^^3^
71 parameters	Δρ_min_ = −0.73 e Å^−^^3^
2 restraints	Extinction correction: SHELXL-2016/6 (Sheldrick 2015), Fc^*^=kFc[1+0.001xFc^2^λ^3^/sin(2θ)]^-1/4^
Primary atom site location: structure-invariant direct methods	Extinction coefficient: 0.000237 (18)

### Thallium digallium arsenic(V) hexakis[hydrogen arsenate(V)] (TlGa2AsHAsO46) Special details

**Table:**

Geometry. All esds (except the esd in the dihedral angle between two l.s. planes) are estimated using the full covariance matrix. The cell esds are taken into account individually in the estimation of esds in distances, angles and torsion angles; correlations between esds in cell parameters are only used when they are defined by crystal symmetry. An approximate (isotropic) treatment of cell esds is used for estimating esds involving l.s. planes.

### Thallium digallium arsenic(V) hexakis[hydrogen arsenate(V)] (TlGa2AsHAsO46) Fractional atomic coordinates and isotropic or equivalent isotropic displacement parameters (Å^2^)

**Table:**

	*x*	*y*	*z*	*U*_iso_*/*U*_eq_	Occ. (<1)
Tl1A	0.000000	0.000000	0.750000	0.074 (4)	0.329 (3)
Tl1B	0.000000	−0.0480 (7)	0.750000	0.0496 (15)	0.1294 (11)
Tl1C	0.000000	−0.0903 (10)	0.750000	0.0387 (10)	0.0941 (11)
Ga1	0.333333	0.666667	0.75604 (2)	0.00849 (8)	
As2	0.333333	0.666667	0.666667	0.00754 (9)	
As1	−0.44448 (3)	−0.40820 (3)	0.71139 (2)	0.00861 (6)	
O1	0.40173 (19)	−0.46663 (18)	0.68629 (3)	0.0112 (3)	
O2	−0.45247 (19)	−0.27070 (18)	0.73408 (3)	0.0116 (3)	
O3	−0.2297 (2)	−0.2872 (2)	0.69852 (4)	0.0209 (3)	
O4	0.48864 (18)	−0.12491 (19)	0.77863 (3)	0.0117 (3)	
H	−0.176 (5)	−0.351 (5)	0.7005 (8)	0.069 (14)*	

### Thallium digallium arsenic(V) hexakis[hydrogen arsenate(V)] (TlGa2AsHAsO46) Atomic displacement parameters (Å^2^)

**Table:**

	*U*^11^	*U*^22^	*U*^33^	*U*^12^	*U*^13^	*U*^23^
Tl1A	0.056 (4)	0.056 (4)	0.109 (4)	0.028 (2)	0.000	0.000
Tl1B	0.061 (2)	0.029 (3)	0.069 (2)	0.0305 (10)	0.0008 (16)	0.0004 (8)
Tl1C	0.0285 (14)	0.030 (3)	0.0566 (17)	0.0142 (7)	0.0073 (11)	0.0037 (5)
Ga1	0.00891 (11)	0.00891 (11)	0.00765 (16)	0.00446 (5)	0.000	0.000
As2	0.00849 (13)	0.00849 (13)	0.00563 (19)	0.00425 (6)	0.000	0.000
As1	0.00960 (10)	0.00911 (10)	0.00843 (9)	0.00564 (7)	0.00016 (6)	0.00061 (6)
O1	0.0147 (6)	0.0116 (6)	0.0096 (6)	0.0082 (5)	−0.0029 (5)	−0.0001 (5)
O2	0.0112 (6)	0.0109 (6)	0.0125 (6)	0.0055 (5)	0.0005 (5)	−0.0023 (5)
O3	0.0137 (7)	0.0206 (8)	0.0291 (9)	0.0092 (6)	0.0089 (6)	0.0085 (7)
O4	0.0133 (6)	0.0105 (6)	0.0136 (6)	0.0077 (5)	−0.0024 (5)	−0.0032 (5)

### Thallium digallium arsenic(V) hexakis[hydrogen arsenate(V)] (TlGa2AsHAsO46) Geometric parameters (Å, º)

**Table:**

Tl1A—Tl1B^i^	0.408 (7)	Tl1B—O4^vii^	3.810 (6)
Tl1A—Tl1B^ii^	0.408 (6)	Tl1B—As1^iv^	3.936 (3)
Tl1A—Tl1C	0.766 (9)	Tl1B—As1^i^	3.936 (3)
Tl1A—Tl1C^i^	0.766 (9)	Tl1C—Tl1C^i^	1.327 (15)
Tl1A—Tl1C^ii^	0.766 (9)	Tl1C—Tl1C^ii^	1.327 (15)
Tl1A—O3	3.4357 (19)	Tl1C—O2^iv^	2.883 (6)
Tl1A—O3^iii^	3.4358 (19)	Tl1C—O2^i^	2.883 (6)
Tl1A—O3^i^	3.4358 (19)	Tl1C—O3	3.186 (3)
Tl1A—O3^iv^	3.4358 (19)	Tl1C—O3^v^	3.186 (3)
Tl1A—O3^v^	3.4358 (19)	Tl1C—O3^iv^	3.3574 (19)
Tl1A—O3^ii^	3.4358 (19)	Tl1C—O3^i^	3.3574 (19)
Tl1A—O2^v^	3.4419 (15)	Tl1C—O2^v^	3.4432 (18)
Tl1A—O2^iv^	3.4419 (15)	Tl1C—O2	3.4432 (18)
Tl1A—O2^ii^	3.4419 (15)	Tl1C—O4^vi^	3.494 (8)
Tl1A—O2^iii^	3.4419 (14)	Tl1C—O4^vii^	3.494 (8)
Tl1A—O2^i^	3.4419 (14)	Tl1C—As1^iv^	3.802 (3)
Tl1A—O2	3.4419 (15)	Tl1C—As1^i^	3.802 (3)
Tl1A—As1^v^	4.1219 (5)	Tl1C—As1^v^	3.8935 (19)
Tl1A—As1^iii^	4.1219 (5)	Tl1C—As1	3.8936 (19)
Tl1A—As1^iv^	4.1219 (5)	Tl1C—O3^iii^	3.969 (7)
Tl1B—Tl1B^i^	0.706 (11)	Ga1—O4^viii^	1.9609 (14)
Tl1B—Tl1B^ii^	0.706 (11)	Ga1—O4^ix^	1.9609 (15)
Tl1B—Tl1C^i^	1.032 (12)	Ga1—O4^i^	1.9609 (14)
Tl1B—Tl1C^ii^	1.032 (11)	Ga1—O2^x^	1.9648 (14)
Tl1B—O2^iv^	3.134 (5)	Ga1—O2^ii^	1.9648 (15)
Tl1B—O2^i^	3.134 (5)	Ga1—O2^xi^	1.9648 (14)
Tl1B—O3	3.283 (3)	As2—O1^xii^	1.8062 (14)
Tl1B—O3^v^	3.283 (3)	As2—O1^xiii^	1.8062 (14)
Tl1B—O3^i^	3.373 (2)	As2—O1^ix^	1.8062 (14)
Tl1B—O3^iv^	3.373 (2)	As2—O1^xiv^	1.8062 (14)
Tl1B—O2^v^	3.4213 (15)	As2—O1^i^	1.8063 (14)
Tl1B—O2	3.4213 (15)	As2—O1^viii^	1.8063 (14)
Tl1B—O3^iii^	3.709 (5)	As1—O2	1.6641 (14)
Tl1B—O3^ii^	3.709 (5)	As1—O4^v^	1.6672 (14)
Tl1B—O2^ii^	3.810 (6)	As1—O1^xv^	1.7094 (14)
Tl1B—O2^iii^	3.810 (6)	As1—O3	1.7115 (16)
Tl1B—O4^vi^	3.810 (6)		
			
Tl1B^i^—Tl1A—Tl1B^ii^	120.00 (2)	O2^i^—Tl1C—O3^i^	49.88 (5)
Tl1B^i^—Tl1A—Tl1C	120.000 (17)	O3—Tl1C—O3^i^	72.40 (6)
Tl1B^ii^—Tl1A—Tl1C	120.00 (7)	O3^v^—Tl1C—O3^i^	107.87 (7)
Tl1B^i^—Tl1A—Tl1C^i^	0 (2)	O3^iv^—Tl1C—O3^i^	178.8 (3)
Tl1B^ii^—Tl1A—Tl1C^i^	120.00 (2)	Tl1A—Tl1C—O2^v^	83.51 (14)
Tl1C—Tl1A—Tl1C^i^	120.000 (19)	Tl1B^i^—Tl1C—O2^v^	64.1 (2)
Tl1B^i^—Tl1A—Tl1C^ii^	120.000 (17)	Tl1B^ii^—Tl1C—O2^v^	102.9 (3)
Tl1B^ii^—Tl1A—Tl1C^ii^	0.000 (19)	Tl1C^i^—Tl1C—O2^v^	54.51 (14)
Tl1C—Tl1A—Tl1C^ii^	120.00 (5)	Tl1C^ii^—Tl1C—O2^v^	112.64 (13)
Tl1C^i^—Tl1A—Tl1C^ii^	120.000 (17)	O2^iv^—Tl1C—O2^v^	132.5 (2)
Tl1B^i^—Tl1A—O3	129.66 (3)	O2^i^—Tl1C—O2^v^	58.33 (6)
Tl1B^ii^—Tl1A—O3	77.72 (3)	O3—Tl1C—O2^v^	136.75 (5)
Tl1C—Tl1A—O3	64.82 (3)	O3^v^—Tl1C—O2^v^	47.23 (4)
Tl1C^i^—Tl1A—O3	129.66 (3)	O3^iv^—Tl1C—O2^v^	115.03 (5)
Tl1C^ii^—Tl1A—O3	77.72 (3)	O3^i^—Tl1C—O2^v^	64.82 (4)
Tl1B^i^—Tl1A—O3^iii^	64.82 (3)	Tl1A—Tl1C—O2	83.51 (14)
Tl1B^ii^—Tl1A—O3^iii^	77.72 (3)	Tl1B^i^—Tl1C—O2	102.9 (3)
Tl1C—Tl1A—O3^iii^	129.65 (3)	Tl1B^ii^—Tl1C—O2	64.1 (2)
Tl1C^i^—Tl1A—O3^iii^	64.82 (3)	Tl1C^i^—Tl1C—O2	112.64 (14)
Tl1C^ii^—Tl1A—O3^iii^	77.72 (3)	Tl1C^ii^—Tl1C—O2	54.51 (15)
O3—Tl1A—O3^iii^	155.44 (5)	O2^iv^—Tl1C—O2	58.33 (6)
Tl1B^i^—Tl1A—O3^i^	64.82 (3)	O2^i^—Tl1C—O2	132.5 (2)
Tl1B^ii^—Tl1A—O3^i^	129.65 (3)	O3—Tl1C—O2	47.23 (4)
Tl1C—Tl1A—O3^i^	77.72 (3)	O3^v^—Tl1C—O2	136.75 (5)
Tl1C^i^—Tl1A—O3^i^	64.82 (3)	O3^iv^—Tl1C—O2	64.82 (4)
Tl1C^ii^—Tl1A—O3^i^	129.65 (3)	O3^i^—Tl1C—O2	115.03 (5)
O3—Tl1A—O3^i^	68.50 (4)	O2^v^—Tl1C—O2	167.0 (3)
O3^iii^—Tl1A—O3^i^	129.64 (6)	Tl1A—Tl1C—O4^vi^	150.55 (7)
Tl1B^i^—Tl1A—O3^iv^	129.65 (3)	Tl1B^i^—Tl1C—O4^vi^	136.76 (15)
Tl1B^ii^—Tl1A—O3^iv^	64.82 (3)	Tl1B^ii^—Tl1C—O4^vi^	155.25 (6)
Tl1C—Tl1A—O3^iv^	77.72 (3)	Tl1C^i^—Tl1C—O4^vi^	128.52 (6)
Tl1C^i^—Tl1A—O3^iv^	129.65 (3)	Tl1C^ii^—Tl1C—O4^vi^	152.31 (4)
Tl1C^ii^—Tl1A—O3^iv^	64.82 (3)	O2^iv^—Tl1C—O4^vi^	58.91 (14)
O3—Tl1A—O3^iv^	100.69 (6)	O2^i^—Tl1C—O4^vi^	49.76 (12)
O3^iii^—Tl1A—O3^iv^	68.50 (4)	O3—Tl1C—O4^vi^	106.8 (2)
O3^i^—Tl1A—O3^iv^	155.44 (5)	O3^v^—Tl1C—O4^vi^	47.94 (9)
Tl1B^i^—Tl1A—O3^v^	77.72 (3)	O3^iv^—Tl1C—O4^vi^	81.43 (12)
Tl1B^ii^—Tl1A—O3^v^	129.65 (3)	O3^i^—Tl1C—O4^vi^	99.64 (15)
Tl1C—Tl1A—O3^v^	64.82 (3)	O2^v^—Tl1C—O4^vi^	75.36 (9)
Tl1C^i^—Tl1A—O3^v^	77.72 (3)	O2—Tl1C—O4^vi^	116.71 (18)
Tl1C^ii^—Tl1A—O3^v^	129.65 (3)	Tl1A—Tl1C—O4^vii^	150.55 (7)
O3—Tl1A—O3^v^	129.64 (6)	Tl1B^i^—Tl1C—O4^vii^	155.24 (6)
O3^iii^—Tl1A—O3^v^	68.50 (4)	Tl1B^ii^—Tl1C—O4^vii^	136.76 (16)
O3^i^—Tl1A—O3^v^	100.69 (5)	Tl1C^i^—Tl1C—O4^vii^	152.31 (4)
O3^iv^—Tl1A—O3^v^	68.50 (4)	Tl1C^ii^—Tl1C—O4^vii^	128.52 (7)
Tl1B^i^—Tl1A—O3^ii^	77.72 (3)	O2^iv^—Tl1C—O4^vii^	49.76 (12)
Tl1B^ii^—Tl1A—O3^ii^	64.82 (3)	O2^i^—Tl1C—O4^vii^	58.91 (15)
Tl1C—Tl1A—O3^ii^	129.65 (3)	O3—Tl1C—O4^vii^	47.94 (9)
Tl1C^i^—Tl1A—O3^ii^	77.72 (3)	O3^v^—Tl1C—O4^vii^	106.8 (2)
Tl1C^ii^—Tl1A—O3^ii^	64.82 (3)	O3^iv^—Tl1C—O4^vii^	99.64 (15)
O3—Tl1A—O3^ii^	68.50 (4)	O3^i^—Tl1C—O4^vii^	81.43 (12)
O3^iii^—Tl1A—O3^ii^	100.69 (5)	O2^v^—Tl1C—O4^vii^	116.71 (18)
O3^i^—Tl1A—O3^ii^	68.50 (4)	O2—Tl1C—O4^vii^	75.36 (9)
O3^iv^—Tl1A—O3^ii^	129.64 (6)	O4^vi^—Tl1C—O4^vii^	58.90 (15)
O3^v^—Tl1A—O3^ii^	155.44 (6)	Tl1A—Tl1C—As1^iv^	109.57 (12)
Tl1B^i^—Tl1A—O2^v^	38.59 (2)	Tl1B^i^—Tl1C—As1^iv^	125.8 (2)
Tl1B^ii^—Tl1A—O2^v^	153.03 (3)	Tl1B^ii^—Tl1C—As1^iv^	92.58 (19)
Tl1C—Tl1A—O2^v^	83.71 (3)	Tl1C^i^—Tl1C—As1^iv^	133.20 (11)
Tl1C^i^—Tl1A—O2^v^	38.59 (2)	Tl1C^ii^—Tl1C—As1^iv^	84.00 (13)
Tl1C^ii^—Tl1A—O2^v^	153.03 (3)	O2^iv^—Tl1C—As1^iv^	24.19 (3)
O3—Tl1A—O2^v^	127.32 (4)	O2^i^—Tl1C—As1^iv^	117.7 (2)
O3^iii^—Tl1A—O2^v^	76.88 (4)	O3—Tl1C—As1^iv^	86.06 (8)
O3^i^—Tl1A—O2^v^	64.02 (4)	O3^v^—Tl1C—As1^iv^	85.56 (8)
O3^iv^—Tl1A—O2^v^	113.04 (4)	O3^iv^—Tl1C—As1^iv^	26.75 (4)
O3^v^—Tl1A—O2^v^	45.62 (4)	O3^i^—Tl1C—As1^iv^	154.2 (2)
O3^ii^—Tl1A—O2^v^	111.53 (4)	O2^v^—Tl1C—As1^iv^	132.74 (7)
Tl1B^i^—Tl1A—O2^iv^	153.03 (2)	O2—Tl1C—As1^iv^	52.91 (3)
Tl1B^ii^—Tl1A—O2^iv^	83.71 (4)	O4^vi^—Tl1C—As1^iv^	72.63 (12)
Tl1C—Tl1A—O2^iv^	38.59 (3)	O4^vii^—Tl1C—As1^iv^	73.46 (12)
Tl1C^i^—Tl1A—O2^iv^	153.03 (2)	Tl1A—Tl1C—As1^i^	109.57 (12)
Tl1C^ii^—Tl1A—O2^iv^	83.71 (3)	Tl1B^i^—Tl1C—As1^i^	92.58 (18)
O3—Tl1A—O2^iv^	64.02 (4)	Tl1B^ii^—Tl1C—As1^i^	125.8 (2)
O3^iii^—Tl1A—O2^iv^	113.04 (4)	Tl1C^i^—Tl1C—As1^i^	84.00 (12)
O3^i^—Tl1A—O2^iv^	111.53 (4)	Tl1C^ii^—Tl1C—As1^i^	133.20 (11)
O3^iv^—Tl1A—O2^iv^	45.62 (3)	O2^iv^—Tl1C—As1^i^	117.7 (2)
O3^v^—Tl1A—O2^iv^	76.88 (4)	O2^i^—Tl1C—As1^i^	24.19 (3)
O3^ii^—Tl1A—O2^iv^	127.32 (4)	O3—Tl1C—As1^i^	85.56 (8)
O2^v^—Tl1A—O2^iv^	114.671 (18)	O3^v^—Tl1C—As1^i^	86.06 (8)
Tl1B^i^—Tl1A—O2^ii^	38.59 (2)	O3^iv^—Tl1C—As1^i^	154.2 (2)
Tl1B^ii^—Tl1A—O2^ii^	83.71 (3)	O3^i^—Tl1C—As1^i^	26.75 (4)
Tl1C—Tl1A—O2^ii^	153.03 (3)	O2^v^—Tl1C—As1^i^	52.91 (3)
Tl1C^i^—Tl1A—O2^ii^	38.59 (2)	O2—Tl1C—As1^i^	132.74 (7)
Tl1C^ii^—Tl1A—O2^ii^	83.71 (3)	O4^vi^—Tl1C—As1^i^	73.46 (12)
O3—Tl1A—O2^ii^	113.05 (4)	O4^vii^—Tl1C—As1^i^	72.63 (12)
O3^iii^—Tl1A—O2^ii^	64.02 (4)	As1^iv^—Tl1C—As1^i^	140.9 (2)
O3^i^—Tl1A—O2^ii^	76.88 (4)	Tl1A—Tl1C—As1^v^	102.03 (12)
O3^iv^—Tl1A—O2^ii^	127.32 (4)	Tl1B^i^—Tl1C—As1^v^	84.78 (18)
O3^v^—Tl1A—O2^ii^	111.53 (4)	Tl1B^ii^—Tl1C—As1^v^	118.9 (2)
O3^ii^—Tl1A—O2^ii^	45.62 (4)	Tl1C^i^—Tl1C—As1^v^	76.18 (12)
O2^v^—Tl1A—O2^ii^	77.17 (4)	Tl1C^ii^—Tl1C—As1^v^	126.86 (11)
O2^iv^—Tl1A—O2^ii^	167.42 (5)	O2^iv^—Tl1C—As1^v^	107.38 (17)
Tl1B^i^—Tl1A—O2^iii^	83.71 (2)	O2^i^—Tl1C—As1^v^	54.77 (6)
Tl1B^ii^—Tl1A—O2^iii^	38.59 (4)	O3—Tl1C—As1^v^	144.21 (19)
Tl1C—Tl1A—O2^iii^	153.03 (3)	O3^v^—Tl1C—As1^v^	25.57 (3)
Tl1C^i^—Tl1A—O2^iii^	83.71 (2)	O3^iv^—Tl1C—As1^v^	97.93 (5)
Tl1C^ii^—Tl1A—O2^iii^	38.59 (3)	O3^i^—Tl1C—As1^v^	82.32 (4)
O3—Tl1A—O2^iii^	111.53 (4)	O2^v^—Tl1C—As1^v^	25.27 (3)
O3^iii^—Tl1A—O2^iii^	45.62 (3)	O2—Tl1C—As1^v^	162.06 (9)
O3^i^—Tl1A—O2^iii^	127.31 (4)	O4^vi^—Tl1C—As1^v^	52.35 (7)
O3^iv^—Tl1A—O2^iii^	76.88 (4)	O4^vii^—Tl1C—As1^v^	104.36 (18)
O3^v^—Tl1A—O2^iii^	113.04 (4)	As1^iv^—Tl1C—As1^v^	109.44 (10)
O3^ii^—Tl1A—O2^iii^	64.02 (4)	As1^i^—Tl1C—As1^v^	61.81 (4)
O2^v^—Tl1A—O2^iii^	114.671 (18)	Tl1A—Tl1C—As1	102.03 (12)
O2^iv^—Tl1A—O2^iii^	114.671 (18)	Tl1B^i^—Tl1C—As1	118.9 (2)
O2^ii^—Tl1A—O2^iii^	53.93 (5)	Tl1B^ii^—Tl1C—As1	84.78 (19)
Tl1B^i^—Tl1A—O2^i^	83.71 (2)	Tl1C^i^—Tl1C—As1	126.86 (12)
Tl1B^ii^—Tl1A—O2^i^	153.03 (4)	Tl1C^ii^—Tl1C—As1	76.18 (13)
Tl1C—Tl1A—O2^i^	38.59 (3)	O2^iv^—Tl1C—As1	54.77 (6)
Tl1C^i^—Tl1A—O2^i^	83.71 (2)	O2^i^—Tl1C—As1	107.38 (17)
Tl1C^ii^—Tl1A—O2^i^	153.03 (3)	O3—Tl1C—As1	25.56 (3)
O3—Tl1A—O2^i^	76.88 (4)	O3^v^—Tl1C—As1	144.21 (19)
O3^iii^—Tl1A—O2^i^	127.31 (4)	O3^iv^—Tl1C—As1	82.32 (4)
O3^i^—Tl1A—O2^i^	45.62 (3)	O3^i^—Tl1C—As1	97.93 (5)
O3^iv^—Tl1A—O2^i^	111.52 (4)	O2^v^—Tl1C—As1	162.06 (9)
O3^v^—Tl1A—O2^i^	64.02 (4)	O2—Tl1C—As1	25.27 (3)
O3^ii^—Tl1A—O2^i^	113.04 (4)	O4^vi^—Tl1C—As1	104.36 (18)
O2^v^—Tl1A—O2^i^	53.93 (5)	O4^vii^—Tl1C—As1	52.35 (7)
O2^iv^—Tl1A—O2^i^	77.17 (4)	As1^iv^—Tl1C—As1	61.81 (4)
O2^ii^—Tl1A—O2^i^	114.671 (18)	As1^i^—Tl1C—As1	109.44 (10)
O2^iii^—Tl1A—O2^i^	167.42 (5)	As1^v^—Tl1C—As1	155.9 (2)
Tl1B^i^—Tl1A—O2	153.03 (2)	Tl1A—Tl1C—O3^iii^	41.80 (9)
Tl1B^ii^—Tl1A—O2	38.59 (3)	Tl1B^i^—Tl1C—O3^iii^	42.53 (9)
Tl1C—Tl1A—O2	83.71 (3)	Tl1B^ii^—Tl1C—O3^iii^	48.38 (13)
Tl1C^i^—Tl1A—O2	153.03 (2)	Tl1C^i^—Tl1C—O3^iii^	45.67 (7)
Tl1C^ii^—Tl1A—O2	38.59 (3)	Tl1C^ii^—Tl1C—O3^iii^	53.67 (6)
O3—Tl1A—O2	45.61 (3)	O2^iv^—Tl1C—O3^iii^	112.74 (5)
O3^iii^—Tl1A—O2	111.53 (4)	O2^i^—Tl1C—O3^iii^	127.49 (5)
O3^i^—Tl1A—O2	113.04 (4)	O3—Tl1C—O3^iii^	139.3 (2)
O3^iv^—Tl1A—O2	64.02 (4)	O3^v^—Tl1C—O3^iii^	64.35 (8)
O3^v^—Tl1A—O2	127.32 (4)	O3^iv^—Tl1C—O3^iii^	63.07 (9)
O3^ii^—Tl1A—O2	76.88 (4)	O3^i^—Tl1C—O3^iii^	115.92 (18)
O2^v^—Tl1A—O2	167.42 (5)	O2^v^—Tl1C—O3^iii^	70.05 (10)
O2^iv^—Tl1A—O2	53.93 (5)	O2—Tl1C—O3^iii^	99.95 (14)
O2^ii^—Tl1A—O2	114.671 (18)	O4^vi^—Tl1C—O3^iii^	110.35 (4)
O2^iii^—Tl1A—O2	77.17 (5)	O4^vii^—Tl1C—O3^iii^	161.91 (11)
O2^i^—Tl1A—O2	114.671 (18)	As1^iv^—Tl1C—O3^iii^	89.69 (6)
Tl1B^i^—Tl1A—As1^v^	60.345 (3)	As1^i^—Tl1C—O3^iii^	120.32 (10)
Tl1B^ii^—Tl1A—As1^v^	151.344 (7)	As1^v^—Tl1C—O3^iii^	74.64 (6)
Tl1C—Tl1A—As1^v^	67.496 (15)	As1—Tl1C—O3^iii^	125.14 (13)
Tl1C^i^—Tl1A—As1^v^	60.345 (3)	O4^viii^—Ga1—O4^ix^	89.32 (6)
Tl1C^ii^—Tl1A—As1^v^	151.344 (7)	O4^viii^—Ga1—O4^i^	89.32 (6)
O3—Tl1A—As1^v^	125.98 (3)	O4^ix^—Ga1—O4^i^	89.32 (6)
O3^iii^—Tl1A—As1^v^	77.63 (3)	O4^viii^—Ga1—O2^x^	177.75 (6)
O3^i^—Tl1A—As1^v^	78.07 (3)	O4^ix^—Ga1—O2^x^	91.19 (6)
O3^iv^—Tl1A—As1^v^	92.52 (3)	O4^i^—Ga1—O2^x^	88.50 (6)
O3^v^—Tl1A—As1^v^	24.05 (3)	O4^viii^—Ga1—O2^ii^	88.50 (6)
O3^ii^—Tl1A—As1^v^	134.64 (3)	O4^ix^—Ga1—O2^ii^	177.75 (6)
O2^v^—Tl1A—As1^v^	23.26 (2)	O4^i^—Ga1—O2^ii^	91.19 (6)
O2^iv^—Tl1A—As1^v^	92.70 (2)	O2^x^—Ga1—O2^ii^	91.01 (6)
O2^ii^—Tl1A—As1^v^	98.35 (2)	O4^viii^—Ga1—O2^xi^	91.19 (6)
O2^iii^—Tl1A—As1^v^	122.48 (2)	O4^ix^—Ga1—O2^xi^	88.50 (6)
O2^i^—Tl1A—As1^v^	49.80 (3)	O4^i^—Ga1—O2^xi^	177.75 (6)
O2—Tl1A—As1^v^	146.60 (2)	O2^x^—Ga1—O2^xi^	91.01 (6)
Tl1B^i^—Tl1A—As1^iii^	67.496 (3)	O2^ii^—Ga1—O2^xi^	91.01 (6)
Tl1B^ii^—Tl1A—As1^iii^	60.35 (2)	O4^viii^—Ga1—Tl1C^ix^	142.93 (5)
Tl1C—Tl1A—As1^iii^	151.344 (7)	O4^ix^—Ga1—Tl1C^ix^	85.12 (7)
Tl1C^i^—Tl1A—As1^iii^	67.496 (3)	O4^i^—Ga1—Tl1C^ix^	54.06 (6)
Tl1C^ii^—Tl1A—As1^iii^	60.345 (15)	O2^x^—Ga1—Tl1C^ix^	35.00 (5)
O3—Tl1A—As1^iii^	134.64 (3)	O2^ii^—Ga1—Tl1C^ix^	96.95 (7)
O3^iii^—Tl1A—As1^iii^	24.05 (3)	O2^xi^—Ga1—Tl1C^ix^	125.17 (6)
O3^i^—Tl1A—As1^iii^	125.98 (3)	O4^viii^—Ga1—Tl1C^viii^	85.12 (8)
O3^iv^—Tl1A—As1^iii^	77.63 (3)	O4^ix^—Ga1—Tl1C^viii^	54.06 (6)
O3^v^—Tl1A—As1^iii^	92.52 (3)	O4^i^—Ga1—Tl1C^viii^	142.93 (6)
O3^ii^—Tl1A—As1^iii^	78.07 (3)	O2^x^—Ga1—Tl1C^viii^	96.95 (8)
O2^v^—Tl1A—As1^iii^	92.70 (2)	O2^ii^—Ga1—Tl1C^viii^	125.17 (6)
O2^iv^—Tl1A—As1^iii^	122.48 (2)	O2^xi^—Ga1—Tl1C^viii^	35.00 (5)
O2^ii^—Tl1A—As1^iii^	49.80 (2)	Tl1C^ix^—Ga1—Tl1C^viii^	119.488 (4)
O2^iii^—Tl1A—As1^iii^	23.26 (2)	O4^viii^—Ga1—Tl1C^i^	54.06 (6)
O2^i^—Tl1A—As1^iii^	146.60 (2)	O4^ix^—Ga1—Tl1C^i^	142.93 (5)
O2—Tl1A—As1^iii^	98.35 (2)	O4^i^—Ga1—Tl1C^i^	85.13 (7)
As1^v^—Tl1A—As1^iii^	99.284 (8)	O2^x^—Ga1—Tl1C^i^	125.17 (6)
Tl1B^i^—Tl1A—As1^iv^	151.344 (7)	O2^ii^—Ga1—Tl1C^i^	35.00 (5)
Tl1B^ii^—Tl1A—As1^iv^	67.50 (2)	O2^xi^—Ga1—Tl1C^i^	96.95 (7)
Tl1C—Tl1A—As1^iv^	60.345 (15)	Tl1C^ix^—Ga1—Tl1C^i^	119.488 (6)
Tl1C^i^—Tl1A—As1^iv^	151.344 (7)	Tl1C^viii^—Ga1—Tl1C^i^	119.488 (1)
Tl1C^ii^—Tl1A—As1^iv^	67.496 (15)	O4^viii^—Ga1—Tl1B^ix^	143.88 (5)
O3—Tl1A—As1^iv^	78.07 (3)	O4^ix^—Ga1—Tl1B^ix^	82.88 (5)
O3^iii^—Tl1A—As1^iv^	92.52 (3)	O4^i^—Ga1—Tl1B^ix^	55.53 (5)
O3^i^—Tl1A—As1^iv^	134.64 (3)	O2^x^—Ga1—Tl1B^ix^	34.12 (4)
O3^iv^—Tl1A—As1^iv^	24.05 (3)	O2^ii^—Ga1—Tl1B^ix^	99.21 (6)
O3^v^—Tl1A—As1^iv^	77.63 (3)	O2^xi^—Ga1—Tl1B^ix^	123.61 (5)
O3^ii^—Tl1A—As1^iv^	125.98 (3)	Tl1C^ix^—Ga1—Tl1B^ix^	2.61 (6)
O2^v^—Tl1A—As1^iv^	122.48 (2)	Tl1C^viii^—Ga1—Tl1B^ix^	116.94 (6)
O2^iv^—Tl1A—As1^iv^	23.26 (2)	Tl1C^i^—Ga1—Tl1B^ix^	122.10 (5)
O2^ii^—Tl1A—As1^iv^	146.60 (2)	O4^viii^—Ga1—Tl1B^i^	55.53 (5)
O2^iii^—Tl1A—As1^iv^	92.70 (2)	O4^ix^—Ga1—Tl1B^i^	143.88 (5)
O2^i^—Tl1A—As1^iv^	98.35 (2)	O4^i^—Ga1—Tl1B^i^	82.88 (5)
O2—Tl1A—As1^iv^	49.80 (2)	O2^x^—Ga1—Tl1B^i^	123.61 (5)
As1^v^—Tl1A—As1^iv^	99.284 (8)	O2^ii^—Ga1—Tl1B^i^	34.12 (4)
As1^iii^—Tl1A—As1^iv^	99.284 (8)	O2^xi^—Ga1—Tl1B^i^	99.21 (5)
Tl1B^i^—Tl1B—Tl1B^ii^	60.001 (6)	Tl1C^ix^—Ga1—Tl1B^i^	116.94 (6)
Tl1B^i^—Tl1B—Tl1C^i^	10.01 (19)	Tl1C^viii^—Ga1—Tl1B^i^	122.10 (6)
Tl1B^ii^—Tl1B—Tl1C^i^	70.01 (18)	Tl1C^i^—Ga1—Tl1B^i^	2.61 (6)
Tl1B^i^—Tl1B—Tl1C^ii^	70.01 (19)	Tl1B^ix^—Ga1—Tl1B^i^	119.553 (4)
Tl1B^ii^—Tl1B—Tl1C^ii^	10.0 (2)	O4^viii^—Ga1—Tl1B^viii^	82.88 (6)
Tl1C^i^—Tl1B—Tl1C^ii^	80.0 (4)	O4^ix^—Ga1—Tl1B^viii^	55.53 (5)
Tl1B^i^—Tl1B—O2^iv^	161.49 (7)	O4^i^—Ga1—Tl1B^viii^	143.88 (5)
Tl1B^ii^—Tl1B—O2^iv^	108.27 (9)	O2^x^—Ga1—Tl1B^viii^	99.21 (6)
Tl1C^i^—Tl1B—O2^iv^	165.03 (5)	O2^ii^—Ga1—Tl1B^viii^	123.61 (5)
Tl1C^ii^—Tl1B—O2^iv^	98.63 (15)	O2^xi^—Ga1—Tl1B^viii^	34.12 (5)
Tl1B^i^—Tl1B—O2^i^	108.27 (8)	Tl1C^ix^—Ga1—Tl1B^viii^	122.10 (6)
Tl1B^ii^—Tl1B—O2^i^	161.49 (6)	Tl1C^viii^—Ga1—Tl1B^viii^	2.61 (6)
Tl1C^i^—Tl1B—O2^i^	98.63 (14)	Tl1C^i^—Ga1—Tl1B^viii^	116.94 (6)
Tl1C^ii^—Tl1B—O2^i^	165.03 (5)	Tl1B^ix^—Ga1—Tl1B^viii^	119.553 (2)
O2^iv^—Tl1B—O2^i^	86.48 (16)	Tl1B^i^—Ga1—Tl1B^viii^	119.553 (1)
Tl1B^i^—Tl1B—O3	122.35 (9)	O1^xii^—As2—O1^xiii^	92.56 (6)
Tl1B^ii^—Tl1B—O3	91.20 (11)	O1^xii^—As2—O1^ix^	87.44 (6)
Tl1C^i^—Tl1B—O3	125.20 (11)	O1^xiii^—As2—O1^ix^	87.44 (6)
Tl1C^ii^—Tl1B—O3	85.15 (9)	O1^xii^—As2—O1^xiv^	92.56 (6)
O2^iv^—Tl1B—O3	69.19 (9)	O1^xiii^—As2—O1^xiv^	92.56 (6)
O2^i^—Tl1B—O3	83.53 (11)	O1^ix^—As2—O1^xiv^	179.99 (7)
Tl1B^i^—Tl1B—O3^v^	91.20 (10)	O1^xii^—As2—O1^i^	87.44 (6)
Tl1B^ii^—Tl1B—O3^v^	122.35 (9)	O1^xiii^—As2—O1^i^	180.0
Tl1C^i^—Tl1B—O3^v^	85.15 (9)	O1^ix^—As2—O1^i^	92.56 (6)
Tl1C^ii^—Tl1B—O3^v^	125.20 (11)	O1^xiv^—As2—O1^i^	87.44 (6)
O2^iv^—Tl1B—O3^v^	83.53 (11)	O1^xii^—As2—O1^viii^	180.0
O2^i^—Tl1B—O3^v^	69.19 (9)	O1^xiii^—As2—O1^viii^	87.44 (6)
O3—Tl1B—O3^v^	142.5 (2)	O1^ix^—As2—O1^viii^	92.56 (6)
Tl1B^i^—Tl1B—O3^i^	76.72 (10)	O1^xiv^—As2—O1^viii^	87.44 (6)
Tl1B^ii^—Tl1B—O3^i^	113.31 (9)	O1^i^—As2—O1^viii^	92.56 (6)
Tl1C^i^—Tl1B—O3^i^	70.81 (8)	O2—As1—O4^v^	117.11 (7)
Tl1C^ii^—Tl1B—O3^i^	118.40 (16)	O2—As1—O1^xv^	115.20 (7)
O2^iv^—Tl1B—O3^i^	121.79 (16)	O4^v^—As1—O1^xv^	100.55 (7)
O2^i^—Tl1B—O3^i^	48.21 (5)	O2—As1—O3	104.32 (8)
O3—Tl1B—O3^i^	71.03 (6)	O4^v^—As1—O3	111.00 (7)
O3^v^—Tl1B—O3^i^	105.28 (8)	O1^xv^—As1—O3	108.62 (8)
Tl1B^i^—Tl1B—O3^iv^	113.32 (10)	O2—As1—Tl1C^ii^	45.23 (13)
Tl1B^ii^—Tl1B—O3^iv^	76.72 (11)	O4^v^—As1—Tl1C^ii^	117.24 (9)
Tl1C^i^—Tl1B—O3^iv^	118.40 (16)	O1^xv^—As1—Tl1C^ii^	142.08 (9)
Tl1C^ii^—Tl1B—O3^iv^	70.81 (9)	O3—As1—Tl1C^ii^	62.00 (11)
O2^iv^—Tl1B—O3^iv^	48.21 (5)	O2—As1—Tl1C	62.05 (11)
O2^i^—Tl1B—O3^iv^	121.79 (16)	O4^v^—As1—Tl1C	101.81 (13)
O3—Tl1B—O3^iv^	105.28 (8)	O1^xv^—As1—Tl1C	155.50 (11)
O3^v^—Tl1B—O3^iv^	71.03 (6)	O3—As1—Tl1C	53.44 (7)
O3^i^—Tl1B—O3^iv^	169.0 (2)	Tl1C^ii^—As1—Tl1C	19.8 (2)
Tl1B^i^—Tl1B—O2^v^	60.43 (10)	O2—As1—Tl1B^ii^	49.87 (9)
Tl1B^ii^—Tl1B—O2^v^	118.57 (9)	O4^v^—As1—Tl1B^ii^	114.51 (7)
Tl1C^i^—Tl1B—O2^v^	50.84 (13)	O1^xv^—As1—Tl1B^ii^	144.91 (7)
Tl1C^ii^—Tl1B—O2^v^	128.2 (3)	O3—As1—Tl1B^ii^	58.46 (9)
O2^iv^—Tl1B—O2^v^	124.22 (15)	Tl1C^ii^—As1—Tl1B^ii^	4.93 (10)
O2^i^—Tl1B—O2^v^	56.67 (6)	Tl1C—As1—Tl1B^ii^	15.14 (18)
O3—Tl1B—O2^v^	133.64 (8)	O2—As1—Tl1B	58.46 (8)
O3^v^—Tl1B—O2^v^	46.81 (4)	O4^v^—As1—Tl1B	106.50 (9)
O3^i^—Tl1B—O2^v^	64.90 (4)	O1^xv^—As1—Tl1B	151.78 (9)
O3^iv^—Tl1B—O2^v^	115.21 (4)	O3—As1—Tl1B	53.97 (7)
Tl1B^i^—Tl1B—O2	118.57 (11)	Tl1C^ii^—As1—Tl1B	15.00 (18)
Tl1B^ii^—Tl1B—O2	60.43 (12)	Tl1C—As1—Tl1B	5.05 (10)
Tl1C^i^—Tl1B—O2	128.2 (3)	Tl1B^ii^—As1—Tl1B	10.20 (16)
Tl1C^ii^—Tl1B—O2	50.84 (14)	O2—As1—Tl1A	54.77 (5)
O2^iv^—Tl1B—O2	56.67 (6)	O4^v^—As1—Tl1A	111.51 (5)
O2^i^—Tl1B—O2	124.22 (15)	O1^xv^—As1—Tl1A	147.41 (5)
O3—Tl1B—O2	46.81 (4)	O3—As1—Tl1A	54.89 (6)
O3^v^—Tl1B—O2	133.64 (9)	Tl1C^ii^—As1—Tl1A	10.09 (12)
O3^i^—Tl1B—O2	115.21 (4)	Tl1C—As1—Tl1A	10.48 (13)
O3^iv^—Tl1B—O2	64.90 (4)	Tl1B^ii^—As1—Tl1A	5.16 (8)
O2^v^—Tl1B—O2	179.0 (2)	Tl1B—As1—Tl1A	5.42 (9)
Tl1B^i^—Tl1B—O3^iii^	48.40 (6)	O2—As1—Tl1B^i^	55.93 (5)
Tl1B^ii^—Tl1B—O3^iii^	56.62 (5)	O4^v^—As1—Tl1B^i^	113.25 (6)
Tl1C^i^—Tl1B—O3^iii^	52.40 (12)	O1^xv^—As1—Tl1B^i^	145.33 (6)
Tl1C^ii^—Tl1B—O3^iii^	62.37 (15)	O3—As1—Tl1B^i^	52.81 (7)
O2^iv^—Tl1B—O3^iii^	113.67 (4)	Tl1C^ii^—As1—Tl1B^i^	10.77 (13)
O2^i^—Tl1B—O3^iii^	128.31 (4)	Tl1C—As1—Tl1B^i^	11.63 (13)
O3—Tl1B—O3^iii^	147.52 (14)	Tl1B^ii^—As1—Tl1B^i^	6.08 (9)
O3^v^—Tl1B—O3^iii^	66.83 (6)	Tl1B—As1—Tl1B^i^	6.76 (10)
O3^i^—Tl1B—O3^iii^	122.76 (13)	Tl1A—As1—Tl1B^i^	2.50 (3)
O3^iv^—Tl1B—O3^iii^	66.00 (6)	O2—As1—Tl1C^xvi^	92.14 (6)
O2^v^—Tl1B—O3^iii^	73.56 (7)	O4^v^—As1—Tl1C^xvi^	41.91 (5)
O2—Tl1B—O3^iii^	105.70 (10)	O1^xv^—As1—Tl1C^xvi^	82.53 (7)
Tl1B^i^—Tl1B—O3^ii^	56.62 (6)	O3—As1—Tl1C^xvi^	152.91 (6)
Tl1B^ii^—Tl1B—O3^ii^	48.40 (5)	Tl1C^ii^—As1—Tl1C^xvi^	123.42 (8)
Tl1C^i^—Tl1B—O3^ii^	62.37 (15)	Tl1C—As1—Tl1C^xvi^	121.18 (11)
Tl1C^ii^—Tl1B—O3^ii^	52.40 (12)	Tl1B^ii^—As1—Tl1C^xvi^	124.63 (6)
O2^iv^—Tl1B—O3^ii^	128.31 (4)	Tl1B—As1—Tl1C^xvi^	123.45 (8)
O2^i^—Tl1B—O3^ii^	113.67 (4)	Tl1A—As1—Tl1C^xvi^	125.61 (5)
O3—Tl1B—O3^ii^	66.84 (6)	Tl1B^i^—As1—Tl1C^xvi^	128.10 (4)
O3^v^—Tl1B—O3^ii^	147.52 (14)	O2—As1—Tl1C^i^	56.83 (5)
O3^i^—Tl1B—O3^ii^	66.00 (6)	O4^v^—As1—Tl1C^i^	114.55 (6)
O3^iv^—Tl1B—O3^ii^	122.76 (13)	O1^xv^—As1—Tl1C^i^	143.72 (6)
O2^v^—Tl1B—O3^ii^	105.70 (10)	O3—As1—Tl1C^i^	51.27 (7)
O2—Tl1B—O3^ii^	73.56 (7)	Tl1C^ii^—As1—Tl1C^i^	11.61 (14)
O3^iii^—Tl1B—O3^ii^	90.99 (15)	Tl1C—As1—Tl1C^i^	12.76 (15)
Tl1B^i^—Tl1B—O2^ii^	15.14 (2)	Tl1B^ii^—As1—Tl1C^i^	7.28 (9)
Tl1B^ii^—Tl1B—O2^ii^	52.07 (3)	Tl1B—As1—Tl1C^i^	8.14 (10)
Tl1C^i^—Tl1B—O2^ii^	22.47 (14)	Tl1A—As1—Tl1C^i^	4.38 (4)
Tl1C^ii^—Tl1B—O2^ii^	61.7 (2)	Tl1B^i^—As1—Tl1C^i^	1.88 (4)
O2^iv^—Tl1B—O2^ii^	160.34 (12)	Tl1C^xvi^—As1—Tl1C^i^	129.990 (14)
O2^i^—Tl1B—O2^ii^	112.79 (4)	As1^xvii^—O1—As2^xviii^	131.79 (8)
O3—Tl1B—O2^ii^	107.73 (10)	As1^xvii^—O1—Tl1C^vi^	76.09 (10)
O3^v^—Tl1B—O2^ii^	106.34 (9)	As2^xviii^—O1—Tl1C^vi^	131.19 (7)
O3^i^—Tl1B—O2^ii^	72.78 (8)	As1^xvii^—O1—Tl1B^vi^	79.08 (7)
O3^iv^—Tl1B—O2^ii^	118.09 (14)	As2^xviii^—O1—Tl1B^vi^	129.47 (6)
O2^v^—Tl1B—O2^ii^	72.62 (8)	Tl1C^vi^—O1—Tl1B^vi^	3.04 (7)
O2—Tl1B—O2^ii^	106.41 (13)	As1—O2—Ga1^xix^	123.98 (8)
O3^iii^—Tl1B—O2^ii^	57.99 (9)	As1—O2—Tl1C^ii^	110.58 (14)
O3^ii^—Tl1B—O2^ii^	41.51 (7)	Ga1^xix^—O2—Tl1C^ii^	121.99 (11)
Tl1B^i^—Tl1B—O2^iii^	52.07 (4)	As1—O2—Tl1B^ii^	106.18 (10)
Tl1B^ii^—Tl1B—O2^iii^	15.14 (3)	Ga1^xix^—O2—Tl1B^ii^	125.29 (8)
Tl1C^i^—Tl1B—O2^iii^	61.7 (2)	Tl1C^ii^—O2—Tl1B^ii^	4.89 (11)
Tl1C^ii^—Tl1B—O2^iii^	22.47 (15)	As1—O2—Tl1B	97.04 (10)
O2^iv^—Tl1B—O2^iii^	112.79 (4)	Ga1^xix^—O2—Tl1B	130.25 (7)
O2^i^—Tl1B—O2^iii^	160.34 (12)	Tl1C^ii^—O2—Tl1B	16.1 (2)
O3—Tl1B—O2^iii^	106.34 (9)	Tl1B^ii^—O2—Tl1B	11.30 (18)
O3^v^—Tl1B—O2^iii^	107.73 (10)	As1—O2—Tl1A	101.96 (6)
O3^i^—Tl1B—O2^iii^	118.09 (14)	Ga1^xix^—O2—Tl1A	128.28 (6)
O3^iv^—Tl1B—O2^iii^	72.78 (8)	Tl1C^ii^—O2—Tl1A	9.54 (13)
O2^v^—Tl1B—O2^iii^	106.41 (13)	Tl1B^ii^—O2—Tl1A	4.65 (8)
O2—Tl1B—O2^iii^	72.62 (8)	Tl1B—O2—Tl1A	6.80 (10)
O3^iii^—Tl1B—O2^iii^	41.51 (7)	As1—O2—Tl1C	92.68 (12)
O3^ii^—Tl1B—O2^iii^	57.99 (9)	Ga1^xix^—O2—Tl1C	131.46 (7)
O2^ii^—Tl1B—O2^iii^	48.37 (9)	Tl1C^ii^—O2—Tl1C	22.0 (3)
Tl1B^i^—Tl1B—O4^vi^	130.74 (4)	Tl1B^ii^—O2—Tl1C	17.2 (2)
Tl1B^ii^—Tl1B—O4^vi^	153.31 (3)	Tl1B—O2—Tl1C	5.98 (12)
Tl1C^i^—Tl1B—O4^vi^	121.93 (15)	Tl1A—O2—Tl1C	12.78 (14)
Tl1C^ii^—Tl1B—O4^vi^	146.99 (14)	As1—O2—Tl1B^i^	102.86 (6)
O2^iv^—Tl1B—O4^vi^	53.44 (9)	Ga1^xix^—O2—Tl1B^i^	128.59 (6)
O2^i^—Tl1B—O4^vi^	45.18 (8)	Tl1C^ii^—O2—Tl1B^i^	7.87 (11)
O3—Tl1B—O4^vi^	98.07 (15)	Tl1B^ii^—O2—Tl1B^i^	3.37 (6)
O3^v^—Tl1B—O4^vi^	44.48 (7)	Tl1B—O2—Tl1B^i^	9.37 (14)
O3^i^—Tl1B—O4^vi^	93.37 (11)	Tl1A—O2—Tl1B^i^	2.78 (4)
O3^iv^—Tl1B—O4^vi^	76.71 (9)	Tl1C—O2—Tl1B^i^	15.31 (17)
O2^v^—Tl1B—O4^vi^	71.57 (8)	As1—O2—Tl1C^i^	103.50 (6)
O2—Tl1B—O4^vi^	109.39 (13)	Ga1^xix^—O2—Tl1C^i^	128.74 (6)
O3^iii^—Tl1B—O4^vi^	109.27 (4)	Tl1C^ii^—O2—Tl1C^i^	7.09 (10)
O3^ii^—Tl1B—O4^vi^	157.13 (10)	Tl1B^ii^—O2—Tl1C^i^	3.69 (4)
O2^ii^—Tl1B—O4^vi^	144.16 (3)	Tl1B—O2—Tl1C^i^	11.31 (13)
O2^iii^—Tl1B—O4^vi^	144.88 (3)	Tl1A—O2—Tl1C^i^	4.82 (5)
Tl1B^i^—Tl1B—O4^vii^	153.31 (3)	Tl1C—O2—Tl1C^i^	17.21 (19)
Tl1B^ii^—Tl1B—O4^vii^	130.74 (5)	Tl1B^i^—O2—Tl1C^i^	2.03 (4)
Tl1C^i^—Tl1B—O4^vii^	146.99 (13)	As1—O2—Tl1C^xvi^	68.04 (4)
Tl1C^ii^—Tl1B—O4^vii^	121.93 (15)	Ga1^xix^—O2—Tl1C^xvi^	59.62 (4)
O2^iv^—Tl1B—O4^vii^	45.18 (8)	Tl1C^ii^—O2—Tl1C^xvi^	140.65 (6)
O2^i^—Tl1B—O4^vii^	53.44 (9)	Tl1B^ii^—O2—Tl1C^xvi^	137.61 (6)
O3—Tl1B—O4^vii^	44.48 (7)	Tl1B—O2—Tl1C^xvi^	128.34 (12)
O3^v^—Tl1B—O4^vii^	98.07 (15)	Tl1A—O2—Tl1C^xvi^	134.48 (5)
O3^i^—Tl1B—O4^vii^	76.70 (9)	Tl1C—O2—Tl1C^xvi^	122.85 (17)
O3^iv^—Tl1B—O4^vii^	93.37 (11)	Tl1B^i^—O2—Tl1C^xvi^	137.26 (5)
O2^v^—Tl1B—O4^vii^	109.39 (13)	Tl1C^i^—O2—Tl1C^xvi^	139.30 (4)
O2—Tl1B—O4^vii^	71.57 (8)	As1—O3—Tl1C	100.99 (8)
O3^iii^—Tl1B—O4^vii^	157.14 (10)	As1—O3—Tl1B	101.09 (7)
O3^ii^—Tl1B—O4^vii^	109.27 (4)	Tl1C—O3—Tl1B	6.12 (13)
O2^ii^—Tl1B—O4^vii^	144.87 (3)	As1—O3—Tl1C^ii^	91.25 (13)
O2^iii^—Tl1B—O4^vii^	144.16 (3)	Tl1C—O3—Tl1C^ii^	23.2 (3)
O4^vi^—Tl1B—O4^vii^	53.60 (10)	Tl1B—O3—Tl1C^ii^	17.8 (2)
Tl1B^i^—Tl1B—As1^iv^	137.73 (8)	As1—O3—Tl1B^ii^	95.92 (11)
Tl1B^ii^—Tl1B—As1^iv^	88.74 (9)	Tl1C—O3—Tl1B^ii^	17.8 (2)
Tl1C^i^—Tl1B—As1^iv^	143.84 (15)	Tl1B—O3—Tl1B^ii^	12.08 (19)
Tl1C^ii^—Tl1B—As1^iv^	80.08 (13)	Tl1C^ii^—O3—Tl1B^ii^	6.11 (13)
O2^iv^—Tl1B—As1^iv^	23.96 (3)	As1—O3—Tl1A	101.07 (7)
O2^i^—Tl1B—As1^iv^	108.05 (16)	Tl1C—O3—Tl1A	12.57 (15)
O3—Tl1B—As1^iv^	82.58 (7)	Tl1B—O3—Tl1A	6.45 (10)
O3^v^—Tl1B—As1^iv^	82.12 (7)	Tl1C^ii^—O3—Tl1A	12.89 (15)
O3^i^—Tl1B—As1^iv^	145.30 (16)	Tl1B^ii^—O3—Tl1A	6.78 (11)
O3^iv^—Tl1B—As1^iv^	25.63 (4)	As1—O3—Tl1B^i^	105.62 (10)
O2^v^—Tl1B—As1^iv^	128.80 (9)	Tl1C—O3—Tl1B^i^	14.88 (17)
O2—Tl1B—As1^iv^	51.74 (4)	Tl1B—O3—Tl1B^i^	9.25 (14)
O3^iii^—Tl1B—As1^iv^	91.55 (4)	Tl1C^ii^—O3—Tl1B^i^	15.81 (18)
O3^ii^—Tl1B—As1^iv^	123.66 (5)	Tl1B^ii^—O3—Tl1B^i^	10.07 (15)
O2^ii^—Tl1B—As1^iv^	138.66 (12)	Tl1A—O3—Tl1B^i^	4.85 (7)
O2^iii^—Tl1B—As1^iv^	90.32 (5)	As1—O3—Tl1C^i^	109.08 (11)
O4^vi^—Tl1B—As1^iv^	67.91 (8)	Tl1C—O3—Tl1C^i^	17.3 (2)
O4^vii^—Tl1B—As1^iv^	68.66 (8)	Tl1B—O3—Tl1C^i^	12.27 (13)
Tl1B^i^—Tl1B—As1^i^	88.74 (8)	Tl1C^ii^—O3—Tl1C^i^	18.6 (2)
Tl1B^ii^—Tl1B—As1^i^	137.73 (6)	Tl1B^ii^—O3—Tl1C^i^	13.23 (14)
Tl1C^i^—Tl1B—As1^i^	80.08 (12)	Tl1A—O3—Tl1C^i^	8.55 (8)
Tl1C^ii^—Tl1B—As1^i^	143.84 (15)	Tl1B^i^—O3—Tl1C^i^	3.70 (7)
O2^iv^—Tl1B—As1^i^	108.05 (16)	As1^v^—O4—Ga1^xviii^	126.29 (8)
O2^i^—Tl1B—As1^i^	23.96 (3)	As1^v^—O4—Tl1C^xx^	119.50 (8)
O3—Tl1B—As1^i^	82.12 (7)	Ga1^xviii^—O4—Tl1C^xx^	98.90 (5)
O3^v^—Tl1B—As1^i^	82.59 (7)	As1^v^—O4—Tl1B^xx^	120.75 (7)
O3^i^—Tl1B—As1^i^	25.63 (3)	Ga1^xviii^—O4—Tl1B^xx^	99.36 (5)
O3^iv^—Tl1B—As1^i^	145.30 (16)	Tl1C^xx^—O4—Tl1B^xx^	2.65 (6)
O2^v^—Tl1B—As1^i^	51.74 (4)	As1^v^—O4—Tl1A^xvii^	121.89 (6)
O2—Tl1B—As1^i^	128.80 (9)	Ga1^xviii^—O4—Tl1A^xvii^	99.79 (5)
O3^iii^—Tl1B—As1^i^	123.66 (5)	Tl1C^xx^—O4—Tl1A^xvii^	5.17 (7)
O3^ii^—Tl1B—As1^i^	91.55 (4)	Tl1B^xx^—O4—Tl1A^xvii^	2.52 (4)
O2^ii^—Tl1B—As1^i^	90.32 (5)	As1^v^—O4—Tl1B^xvii^	126.95 (10)
O2^iii^—Tl1B—As1^i^	138.66 (12)	Ga1^xviii^—O4—Tl1B^xvii^	96.40 (7)
O4^vi^—Tl1B—As1^i^	68.66 (8)	Tl1C^xx^—O4—Tl1B^xvii^	9.46 (12)
O4^vii^—Tl1B—As1^i^	67.90 (8)	Tl1B^xx^—O4—Tl1B^xvii^	7.14 (11)
As1^iv^—Tl1B—As1^i^	131.02 (16)	Tl1A^xvii^—O4—Tl1B^xvii^	5.24 (8)
Tl1A—Tl1C—Tl1B^i^	19.99 (19)	As1^v^—O4—Tl1C^xvii^	131.11 (13)
Tl1A—Tl1C—Tl1B^ii^	19.99 (19)	Ga1^xviii^—O4—Tl1C^xvii^	93.57 (8)
Tl1B^i^—Tl1C—Tl1B^ii^	40.0 (4)	Tl1C^xx^—O4—Tl1C^xvii^	13.52 (16)
Tl1A—Tl1C—Tl1C^i^	30.0	Tl1B^xx^—O4—Tl1C^xvii^	11.37 (12)
Tl1B^i^—Tl1C—Tl1C^i^	10.01 (19)	Tl1A^xvii^—O4—Tl1C^xvii^	9.57 (10)
Tl1B^ii^—Tl1C—Tl1C^i^	50.00 (19)	Tl1B^xvii^—O4—Tl1C^xvii^	4.33 (9)
Tl1A—Tl1C—Tl1C^ii^	30.001 (5)	As1^v^—O4—Tl1B^xxi^	117.93 (8)
Tl1B^i^—Tl1C—Tl1C^ii^	50.00 (19)	Ga1^xviii^—O4—Tl1B^xxi^	103.39 (7)
Tl1B^ii^—Tl1C—Tl1C^ii^	10.01 (19)	Tl1C^xx^—O4—Tl1B^xxi^	5.57 (7)
Tl1C^i^—Tl1C—Tl1C^ii^	60.001 (5)	Tl1B^xx^—O4—Tl1B^xxi^	4.08 (6)
Tl1A—Tl1C—O2^iv^	131.87 (13)	Tl1A^xvii^—O4—Tl1B^xxi^	4.01 (6)
Tl1B^i^—Tl1C—O2^iv^	149.7 (2)	Tl1B^xvii^—O4—Tl1B^xxi^	9.04 (13)
Tl1B^ii^—Tl1C—O2^iv^	113.0 (2)	Tl1C^xvii^—O4—Tl1B^xxi^	13.33 (14)
Tl1C^i^—Tl1C—O2^iv^	157.37 (11)	As1^v^—O4—Tl1C	57.11 (10)
Tl1C^ii^—Tl1C—O2^iv^	103.48 (14)	Ga1^xviii^—O4—Tl1C	69.38 (10)
Tl1A—Tl1C—O2^i^	131.87 (13)	Tl1C^xx^—O4—Tl1C	132.52 (6)
Tl1B^i^—Tl1C—O2^i^	113.0 (2)	Tl1B^xx^—O4—Tl1C	135.13 (5)
Tl1B^ii^—Tl1C—O2^i^	149.7 (2)	Tl1A^xvii^—O4—Tl1C	137.61 (4)
Tl1C^i^—Tl1C—O2^i^	103.48 (13)	Tl1B^xvii^—O4—Tl1C	140.03 (5)
Tl1C^ii^—Tl1C—O2^i^	157.37 (11)	Tl1C^xvii^—O4—Tl1C	141.67 (5)
O2^iv^—Tl1C—O2^i^	96.3 (3)	Tl1B^xxi^—O4—Tl1C	137.05 (4)
Tl1A—Tl1C—O3	102.61 (15)	As1^v^—O4—Tl1C^xxi^	114.85 (9)
Tl1B^i^—Tl1C—O3	112.72 (18)	Ga1^xviii^—O4—Tl1C^xxi^	106.19 (8)
Tl1B^ii^—Tl1C—O3	91.37 (17)	Tl1C^xx^—O4—Tl1C^xxi^	7.53 (9)
Tl1C^i^—Tl1C—O3	116.99 (14)	Tl1B^xx^—O4—Tl1C^xxi^	6.86 (7)
Tl1C^ii^—Tl1C—O3	85.65 (15)	Tl1A^xvii^—O4—Tl1C^xxi^	7.13 (7)
O2^iv^—Tl1C—O3	73.65 (11)	Tl1B^xvii^—O4—Tl1C^xxi^	12.10 (13)
O2^i^—Tl1C—O3	89.44 (14)	Tl1C^xvii^—O4—Tl1C^xxi^	16.34 (17)
Tl1A—Tl1C—O3^v^	102.61 (15)	Tl1B^xxi^—O4—Tl1C^xxi^	3.13 (6)
Tl1B^i^—Tl1C—O3^v^	91.37 (16)	Tl1C—O4—Tl1C^xxi^	136.41 (4)
Tl1B^ii^—Tl1C—O3^v^	112.72 (18)	As1^v^—O4—Tl1B	53.77 (7)
Tl1C^i^—Tl1C—O3^v^	85.65 (14)	Ga1^xviii^—O4—Tl1B	72.86 (7)
Tl1C^ii^—Tl1C—O3^v^	116.99 (13)	Tl1C^xx^—O4—Tl1B	131.57 (7)
O2^iv^—Tl1C—O3^v^	89.44 (14)	Tl1B^xx^—O4—Tl1B	134.21 (4)
O2^i^—Tl1C—O3^v^	73.65 (11)	Tl1A^xvii^—O4—Tl1B	136.72 (4)
O3—Tl1C—O3^v^	154.8 (3)	Tl1B^xvii^—O4—Tl1B	139.57 (6)
Tl1A—Tl1C—O3^iv^	89.40 (15)	Tl1C^xvii^—O4—Tl1B	141.61 (6)
Tl1B^i^—Tl1C—O3^iv^	101.8 (2)	Tl1B^xxi^—O4—Tl1B	135.80 (4)
Tl1B^ii^—Tl1C—O3^iv^	77.01 (17)	Tl1C—O4—Tl1B	3.71 (8)
Tl1C^i^—Tl1C—O3^iv^	107.76 (14)	Tl1C^xxi^—O4—Tl1B	134.90 (4)
Tl1C^ii^—Tl1C—O3^iv^	71.13 (14)	As1^v^—O4—Tl1C^i^	44.77 (8)
O2^iv^—Tl1C—O3^iv^	49.88 (5)	Ga1^xviii^—O4—Tl1C^i^	83.27 (9)
O2^i^—Tl1C—O3^iv^	131.2 (2)	Tl1C^xx^—O4—Tl1C^i^	124.50 (15)
O3—Tl1C—O3^iv^	107.87 (7)	Tl1B^xx^—O4—Tl1C^i^	127.13 (11)
O3^v^—Tl1C—O3^iv^	72.40 (6)	Tl1A^xvii^—O4—Tl1C^i^	129.62 (9)
Tl1A—Tl1C—O3^i^	89.39 (15)	Tl1B^xvii^—O4—Tl1C^i^	133.50 (7)
Tl1B^i^—Tl1C—O3^i^	77.01 (16)	Tl1C^xvii^—O4—Tl1C^i^	136.55 (3)
Tl1B^ii^—Tl1C—O3^i^	101.8 (2)	Tl1B^xxi^—O4—Tl1C^i^	127.83 (11)
Tl1C^i^—Tl1C—O3^i^	71.13 (14)	Tl1C—O4—Tl1C^i^	16.03 (19)
Tl1C^ii^—Tl1C—O3^i^	107.76 (13)	Tl1C^xxi^—O4—Tl1C^i^	126.30 (13)
O2^iv^—Tl1C—O3^i^	131.2 (2)	Tl1B—O4—Tl1C^i^	12.41 (14)

Symmetry codes: (i) −*y*, *x*−*y*, *z*; (ii) −*x*+*y*, −*x*, *z*; (iii) *x*−*y*, −*y*, −*z*+3/2; (iv) *y*, *x*, −*z*+3/2; (v) −*x*, −*x*+*y*, −*z*+3/2; (vi) −*y*, *x*−*y*−1, *z*; (vii) *y*, *x*−1, −*z*+3/2; (viii) −*x*+*y*+1, −*x*+1, *z*; (ix) *x*, *y*+1, *z*; (x) −*y*, *x*−*y*+1, *z*; (xi) *x*+1, *y*+1, *z*; (xii) *x*−*y*−1/3, *x*+1/3, −*z*+4/3; (xiii) *y*+2/3, −*x*+*y*+4/3, −*z*+4/3; (xiv) −*x*+2/3, −*y*+1/3, −*z*+4/3; (xv) *x*−1, *y*, *z*; (xvi) −*y*−1, *x*−*y*−1, *z*; (xvii) *x*+1, *y*, *z*; (xviii) *x*, *y*−1, *z*; (xix) *x*−1, *y*−1, *z*; (xx) −*x*+*y*+1, −*x*, *z*; (xxi) −*y*+1, *x*−*y*, *z*.

### Thallium digallium arsenic(V) hexakis[hydrogen arsenate(V)] (TlGa2AsHAsO46) Hydrogen-bond geometry (Å, º)

**Table:**

*D*—H···*A*	*D*—H	H···*A*	*D*···*A*	*D*—H···*A*
O3—H···O4^vii^	0.87 (2)	1.94 (3)	2.728 (2)	150 (4)

Symmetry code: (vii) *y*, *x*−1, −*z*+3/2.
